# The diverse effects of phenotypic dominance on hybrid fitness

**DOI:** 10.1111/evo.14645

**Published:** 2022-10-27

**Authors:** Hilde Schneemann, Aslı D. Munzur, Ken A. Thompson, John J. Welch

**Affiliations:** ^1^ Department of Genetics University of Cambridge Downing Street Cambridge UK; ^2^ Department of Zoology & Biodiversity Research Centre University of British Columbia Vancouver Canada; ^3^ Current address: Department of Biology, Stanford University & Department of Plant Biology Carnegie Institution for Science Stanford USA

**Keywords:** Darwin's corollary, Fisher's geometric model, heterosis, haldane's rule, optimal outbreeding, speciation

## Abstract

When divergent populations interbreed, their alleles are brought together in hybrids. In the initial F1 cross, most divergent loci are heterozygous. Therefore, F1 fitness can be influenced by dominance effects that could not have been selected to function well together. We present a systematic study of these F1 dominance effects by introducing variable phenotypic dominance into Fisher's geometric model. We show that dominance often reduces hybrid fitness, which can generate optimal outbreeding followed by a steady decline in F1 fitness, as is often observed. We also show that “lucky” beneficial effects sometimes arise by chance, which might be important when hybrids can access novel environments. We then show that dominance can lead to violations of Haldane's Rule (reduced fitness of the heterogametic F1) but strengthens Darwin's Corollary (F1 fitness differences between cross directions). Taken together, results show that the effects of dominance on hybrid fitness can be surprisingly difficult to isolate, because they often resemble the effects of uniparental inheritance or expression. Nevertheless, we identify a pattern of environment‐dependent heterosis that only dominance can explain, and for which there is some suggestive evidence. Our results also show how existing data set upper bounds on the size of dominance effects. These bounds could explain why additive models often provide good predictions for later‐generation recombinant hybrids, even when dominance qualitatively changes outcomes for the F1.

During hybridization, alleles from diverged genomes can be expressed together for the first time. The interactions between these alleles will help to determine the outcome of the hybridization. If hybrids are sufficiently fit, for example, then the hybridization might lead to ongoing gene flow (e.g. Lee et al., 2013; Rieseberg et al., 1999), or to a hybrid swarm, potentially adapted to a new niche (e.g. Taylor et al., 2006). If hybrids are unfit, by contrast, the lineages will remain reproductively isolated, and may be subject to reinforcement selection. All hybrids trace their ancestry to a first‐generation, or “F1” hybrid, and so the fitness of the F1 has a special importance. Ongoing gene flow, for example, is only possible if the F1 are both viable and fertile (Blair, 1955; Butlin, 1987; Dobzhansky, 1937; Fisher, 1930; Wallace, 1889). Moreover, the F1 are often easier to form than later generation crosses (Muller, 1940; Vetukhiv, 1954), and so have been more extensively studied.

Decades of data on F1 fitness show that all possible patterns are sometimes observed. In some cases, as illustrated in Figure 1a, the F1 are fitter than both of their parents (best‐parent heterosis); while in other cases, the F1 are less fit (Fig. 1b), or intermediate (Fig. 1c; Arnold & Hodges, 1995; Coyne & Orr, 2004; Wellhausen, 1952; Edmands, 2007; Hatfield & Schluter, 1999; Fraïsse et al., 2016, Table S1; Coughlan & Matute, 2020; Favre et al., 2017; Gramlich et al., 2022; Thompson & Schluter, 2022). Moreover, as illustrated in Figure 1d, the direction of the cross may be important, with strong fitness differences between the reciprocal F1 (i.e., female‐male vs. male‐female cross directions of the same parental lines). This pattern, known as “Darwin's Corollary” (Kölreuters, 1766; Darwin, 1859, Ch. 8; Turelli & Moyle, 2007) is very widely observed in plant (Tiffin et al., 2001), fungus (Dettman et al., 2003), and animal systems (Bolnick et al., 2008; Brandvain et al., 2014), including simultaneous hermaphrodites (Bouchemousse et al., 2016; Escobar et al., 2008; Fraïsse et al., 2016; Sato et al., 2014). Finally, the outcomes are often environment‐dependent, with hybrids fitter than parents in some conditions, and less fit in others (e.g. Wang et al., 1997).

**Figure 1:**
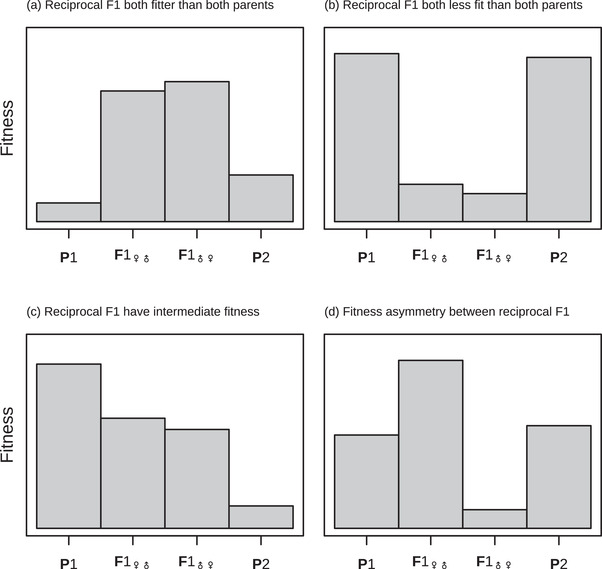
A cartoon illustration of some commonly observed patterns in F1 hybrid fitness. Shown are hypothetical fitness measurements for two parental lines, P1 and P2, and the reciprocal F1 (i.e., the female‐male and male‐female cross of the two lines). **(a)** Both reciprocal F1 being fitter than both parents (best‐parent heterosis) can be explained without phenotypic dominance, unless the parents are phenotypically identical for the relevant traits (see row 1 Table 1). **(b)** Both reciprocal F1 being less fit than both parents can be explained either by phenotypic dominance or uniparental effects (see row 2 Table 1). Observing patterns **(a)** and **(b)** for the same cross in different environments, is a telltale sign of dominance (see row 8 Table 1). **(c)** F1 with intermediate fitness compared to their parents can be explained under any version of the model. **(d)** Fitness differences between the F1 cross directions (the two middle bars) implies the presence of uniparental inheritance or expression of some determinants of the phenotype (see row 7 Table 1).

Despite this heterogeneity, two factors have been shown to predict the fitness of F1 hybrids with reasonable consistency. The first factor is the level of genetic divergence between the parents. Patterns like Fig. 1a are most often observed with closely related lines, but with the level of heterosis increasing with divergence (Birchler, 2013). By contrast, Fig. 1b is most commonly observed between more distantly related lines, with F1 fitness often decreasing steadily with divergence, and so forming an “F1 clock” (Coughlan & Matute, 2020; Edmands, 2002). The combined result is a pattern of “optimal outbreeding,” where the F1 are fittest between parents of intermediate genetic distance (Bateson, 1978; Butlin, 1987; Dagilis et al., 2019; Waser, 1993; Wei & Zhang, 2018).

A second key factor that often predicts the fitness of F1 hybrids is their sex, such that one sex is as fit or fitter than the parents (Fig. 1a), while the other sex is inviable or infertile (Fig. 1b). When such a difference is observed, and sex chromosomes are heteromorphic, the heterogametic sex is almost always inferior to the homogametic sex. This pattern is well‐known as Haldane's Rule (Haldane, 1922; Coyne & Orr, 2004, Ch. 8; Schilthuizen et al., 2011).

What remains unclear is whether these disparate patterns in F1 fitness needs to be explained in a piecemeal way, or whether some common properties of gene interaction—shared by disparate animals, plants and fungi—might help to explain them all. Suggestive of the second possibility, a class of fitness landscapes based on Fisher's geometric model (Fisher, 1930; Orr, 1998) has been successful in generating many of these empirical patterns (Barton, 2001; Chevin et al., 2014; Fraïsse et al., 2016; Mani & Clarke, 1990; Simon et al., 2018; Schneemann et al., 2020). However, there are also reasons to be suspicious. In particular, while the model always allows for dominance in fitness, most studies have assumed phenotypic additivity—that is, they assume that the phenotypic effect of a homozygous allele is exactly double its heterozygous effect. By contrast, empirical studies indicate that variable levels of phenotypic dominance are common for loci affecting quantitative traits, including fitness components (Lynch & Walsh, 1998, p.485; Clo et al., 2021). Given its high heterozygosity, the F1 will be especially affected by this variable dominance. There are, moreover, reasons to expect dominance effects to evolve in a qualitatively different way from additive effects. Additive effects act together in homozygous genotypes, and so they will often be co‐adapted—that is, selected to function well together in the parental environments. By contrast, the dominance effects will act together only in early generation hybrids, and so lack any tendency to be co‐adapted.

Here, building on previous work (Schneemann et al., 2020), we systematically explore the effects of variable phenotypic dominance on hybrid fitness, with a particular focus on the F1. Our global aim, as summarized in Table 1, is to ask how various empirically observed patterns are affected by phenotypic dominance, and whether they provide evidence for, or against, its importance for hybrid fitness. We find that some of the well‐established patterns provide upper bounds on the typical size of dominance effects in nature, while not ruling out their presence altogether. Other patterns have multiple possible explanations, making it difficult to establish their true cause(s). Nevertheless, we identify a single pattern that only dominance can explain.

**Table 1:** Which patterns in F1 fitness can be generated by which model

			Strictly biparental inheritance/expression		Some uniparental inheritance/expression	
			Without	With	Without	With
			phenotypic	phenotypic	phenotypic	phenotypic
	Observation	Section	dominance	dominance	dominance	dominance
1.	Reciprocal F1 both fitter than both parents	1, 2.2, 3, Fig 1a	✓ ^1^	✓	✓ ^1^	✓
2.	Reciprocal F1 both less fit than both parents	2, 4.1, Fig 1b	✗	✓	✓	✓
3.	Optimal outbreeding and F1 clock	2.1, 4.1	✗	✓	✓	✓
4.	Reciprocal F1 of locally adapted parents both fitter in one parental environment	3.2	✓ ^2^	✓	✓ ^2^	✓
5.	Homogametic F1 fitter than heterogametic F1 (Haldane's rule)	4.2	–	–	✓	✓ ^3^
6.	Heterogametic F1 fitter than homogametic F1 (Anti‐Haldane's rule)	4.2	–	–	✓ ^4^	✓ ^5^
7.	Fitness asymmetry between reciprocal F1 (Darwin's corollary)	4.3, Fig 1d	✗	✗	✓ ^6^	✓
8.	Reciprocal F1 both fitter than both parents in one environment, and less fit in another	3.1, Fig 1a *and* Fig 1b	✗	✓	✗	✓

*Note*: 1. Only if parents are phenotypically distinct; 2. Only if parental environments have different selection regimes; 3. Only if $V_{\delta }<\left(1-x\right)$, where *x* is the proportion of divergent sites that are uniparentally inherited; 4. Only with X silencing in females (or Z silencing in males); 5. Only if $V_{\delta }>\left(1-x\right)$ (see main text); 6. Increasingly unlikely at high divergence, *d*.

## Model

Fisher's (1930) geometric model assigns fitnesses to genotypes using a simple model of *n* quantitative traits under optimizing selection. The parameter *n*, which is sometimes called “organismal complexity,” is also the dimensionality of the fitness landscape. An individual's phenotype is represented by a point in this landscape, with its trait values collected in the *n*‐dimensional vector, z. The relative fitness of the individual is denoted as *w*, and depends on the Euclidean distance of the phenotype from some optimum o, whose position is determined by the current environmental conditions. Here, we will use a simple quadratic model, where log fitness declines with the squared distance to the optimum:

(1) $$\begin{array}{cc} \ln w & =-{\left\|z-o\right\|}^{2} \end{array}$$

(2) $$\begin{array}{cc} & \equiv -\sum_{i=1}^{n}{\left(z_{i}-o_{i}\right)}^{2} \end{array}$$

**Figure 2:**
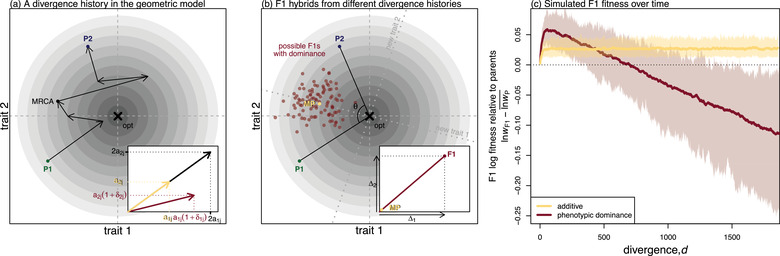
The effects of variable phenotypic dominance on F1 hybrid phenotypes and fitness in Fisher's geometric model. **(a** **and b)** illustrations of the fitness landscape model with n=2 traits for visualisation. Contour lines indicate fitness, with darker colors closer to the optimum (× symbol). : A pair of parental lines, P1 and P2, connected by a chain of d=6 fixed differences. These pass through their most recent common ancestor (MRCA), but are orientated from P1 to P2. The inset shows a single substitution in homozygous state (black arrow), and in heterozygous state either with or without dominance. With additivity (yellow arrow), the heterozygous substitution is simply half of the homozygous substitution. With dominance (dark red arrow), the heterozygous effect on trait *i* is $a_{ij}\left(1+\delta_{ij}\right)$, where $a_{ij}$ is the additive effect, and $\delta_{ij}$ the dominance coefficient. **(b)** Parents and possible F1 hybrids. The yellow point (MP) shows the midparental phenotype located halfway between the parents, which is equivalent to the F1 under strictly biparental inheritance and additivity. Phenotypic dominance pushes the F1 phenotype away from the midparent. Dark red points show a cloud of possible F1 that could have resulted from different realisations of the divergence history. The inset panel shows the total dominance deviation on each trait ($\Delta_{i}$, Equation 4), and dotted lines indicate the new axes after the rotation implied by Equation 5. **(c)** Simulation results illustrating the difference between F1 and parental log fitness over the course of divergence. Under additivity (yellow lines), the F1 maintains a roughly constant level of heterosis. With phenotypic dominance (dark red lines) the F1 shows a pattern of optimal outbreeding with an initial spike in F1 fitness followed by a steady decline (the F1 clock). Lines show the means across 100 replicate simulations and the shading shows 95% quantiles. The simulation procedure assumed that larger effect mutations were more likely to be recessive (Fig. S1), and is described in full in Appendix 2. Parameters were n=20 and $U=\bar{s}=1/N=0.01$.

Substitutions are modeled as *n*‐dimensional vectors of change to the phenotype. Thus, any two phenotypes can be connected by a chain of vectors that represent the divergent alleles accrued since their most recent common ancestor. This is illustrated in Figure 2a, where we show a chain of d=6 genomic differences connecting two homozygous parental lines, labelled P1 and P2. In the illustration, each population fixed equal numbers of substitutions, but this is not an assumption of the analyses. What is important, however, is that all substitution effects are defined relative to the P1 genotype. This means that none of our results depend on knowing whether the P1 alleles are ancestral or derived.

Any given hybrid between P1 and P2 will contain some combination of the *d* vectors. Under phenotypic additivity, the effects of these vectors sum together both between and within loci. This means that an allele carried as a heterozygote will have the same orientation, but half the length, of the allele carried as a homozygote. This is shown by the yellow arrow in Figure 2a inset. To model phenotypic dominance, we relax the assumption of additivity within loci, using dominance coefficients $\delta_{ij}$. This is shown by the dark red arrow in Figure 2a inset. Non‐zero values of the $\delta_{ij}$ can alter both the length of the vector, and its orientation if different traits show different levels of dominance.

We note that the very simple model described above can be derived, either exactly or approximately, from a large family of more complex fitness functions (Martin, 2014; Martin & Lenormand, 2006). In that case, few if any of the *n* traits correspond to real quantitative traits, so that *n* becomes a phenomenological parameter of the fitness landscape (Martin & Lenormand, 2006; Orr, 2000; Welch & Waxman, 2003).

## Results

To explore the various effects of dominance on the fitness of F1 hybrids, we consider several different scenarios and patterns. As such, throughout the results section, we present the major results without derivation, and relegate the full derivations to Appendix 1. Simulations are used solely to illustrate the analytical results.

### THE F1 UNDER ADDITIVITY AND BIPARENTAL INHERITANCE

Let us start with a simple model of strictly biparental inheritance and expression. Although such a model cannot generate Haldane's Rule or Darwin's Corollary (rows 5–7 Table 1), it allows us to isolate the effects of dominance from other factors.

With strictly biparental inheritance, F1 hybrids contain all *d* of the divergent alleles as heterozygotes, and so under phenotypic additivity the F1 phenotype matches the midparental phenotype, obtained by averaging the parental values for all *n* traits. This midparent is illustrated by the yellow point labelled MP in Figure 2b. The fitness of the midparental phenotype, *w*_MP_, therefore depends solely on the fitnesses of the parental lines, *w*_P1_ and *w*_P2_, and their relative positions in the *n*‐dimensional phenotypic space. This is characterized by θ, the angle in radians between the parental phenotypes (see Fig. 2b). Indeed, from the definition of cosine similarity, we find:

(3) $$\begin{array}{cc} \ln w_{\mathrm{MP}} & =-\sum_{i=1}^{n}{\left(\frac{z_{P1,i}+z_{P2,i}}{2}-o_{i}\right)}^{2}\\ & =\frac{\bar{\ln w_{P}}+{\bar{\ln w_{P}}}^{\left(g\right)}\cos\theta }{2} \end{array}$$

where $\bar{\ln w_{P}}\equiv \frac{1}{2}\left(\ln w_{P1}+\ln w_{P2}\right)$ and ${\bar{\ln w_{P}}}^{\left(g\right)}\equiv -\sqrt{\left(\ln w_{P1}\times \ln w_{P2}\right)}$ are respectively the arithmetic and geometric mean log fitness of the parents. Since cosθ varies between −1 and 1, it follows that $\ln w_{\mathrm{MP}}\geq \bar{\ln w_{P}}$. Therefore, with phenotypic additivity, the F1 will always be at least as fit as the average of the fitnesses of the two parental lines (Barton, 2001; Fraïsse et al., 2016). Moreover, as long as the parental lines are not phenotypically identical, F1 fitness may exceed that of both parents, generating the pattern shown in Fig. 1a (row 1 Table 1).

To understand the consequences of this result, let us consider a concrete situation, in which the two parental lines remain well adapted to a fixed phenotypic optimum (effective stabilizing selection) but diverge from their common ancestor largely via “system drift” (Schiffman & Ralph, 2021; Schneemann et al., 2020). In the initial stages of divergence the parental lines fix different alleles, and so they wander slightly in phenotypic space while remaining near the optimum. This wandering tends to increase θ, bringing the midparent closer to the optimum and thereby increasing the potential for heterosis (Equation 3). After this initial divergence, heterosis remains at roughly constant levels because selection prevents the parental lines from wandering any further and hence the phenotypic distance between them has no further tendency to increase. This is confirmed by individual‐based simulations of Fisher's geometric model, which are described in detail in Appendix 2 and shown by the yellow lines in Figure 2c. With a fixed optimum and additive phenotypes, F1 heterosis shows a brief initial increase before settling at a constant positive value.

This prediction of heterosis at all levels of genetic divergence implies that, with additivity and strictly biparental inheritance, the pattern shown in Figure 1b could never arise, and so neither could the patterns of optimal outbreeding or the F1 clock (see rows 2–3 Table 1). Furthermore, even if we considered a different type of fitness landscape in which fitness declines more rapidly with distance to the optimum, this could lead to loss of midparental heterosis, but the F1 could never have lower fitness than both of its parents.

### THE DELETERIOUS EFFECTS OF PHENOTYPIC DOMINANCE

The principal effect of variable phenotypic dominance is to push the F1 phenotype away from the midparent, usually reducing its fitness (Schneemann et al., 2020). To see this, let us first define the total dominance deviation on trait *i*, including contributions from all *d* substitutions.

(4) $$\Delta_{i}\equiv \sum_{j=1}^{d}a_{ij}\delta_{ij}$$

Here, $a_{ij}$ is the additive effect of substitution *j* on trait *i*, and $\delta_{ij}$ is its dominance coefficient. These quantities are illustrated in the inset panels in Figure 2a and 2b. With this definition, the fitness of the F1 can be written as:

(5) $$\ln w_{F1}=-{\left(\sqrt{|\ln w_{\mathrm{MP}}|}+\Delta_{1}\right)}^{2}-\sum_{i=2}^{n}\Delta_{i}^{2}$$

Here, without loss of generality, we have rotated the trait axes such that the midparent falls short of the optimum only for trait 1 (as newly defined), but is optimal for the remaining n−1 traits. This is shown in Figure 2b.

An important point about Equation 5 is that the joint effects of dominance across divergent sites, the $\Delta_{i}$, are first expressed in hybrids. This is because the heterozygous effect $a_{ij}\delta_{ij}$ of an allele at one locus is only expressed during its segregation, and becomes hidden from selection once it fixes. So, whereas the additive effects are expressed jointly in the P2 genotype, the dominance effects are unlikely to be under selection together during divergence, especially allopatric divergence. As such, the joint effects of dominance across divergent sites, captured by the $\Delta_{i}$ will show no tendency to be co‐adapted or remain close to any optimum, even when selection acts on the $\delta_{ij}$ individually. This means that similar scenarios of parental divergence, with similar trajectories for the parental phenotypes, can nonetheless yield very different $\Delta_{i}$ and thus very different F1 phenotypes. This is illustrated by the cloud of dark red points in Figure 2b, which show possible F1 that might have resulted from the same divergence scenario between P1 and P2.

To predict F1 fitness, we must average over these possible evolutionary histories. Let us first consider the scenario discussed above, where parental populations remain well adapted to the same constant environment. In this case, we can think of the $\Delta_{i}$ as undergoing a random walk away from the midparent, so that the set of possible F1 form a growing cloud (Schneemann et al., 2020). This yields:

(6) $$\begin{array}{cc} E\left(\ln w_{F1}\right) & =E\left(\ln w_{\mathrm{MP}}\right)-nV_{\Delta } \end{array}$$

(7) $$\begin{array}{cc} & \;=\frac{\bar{\ln w_{P}}}{2}-dnV_{a\delta } \end{array}$$

where $V_{\Delta }$ is the variance, across divergence histories, of the the total dominance deviations (see Equations 4 and 27); while $V_{a\delta }$ is the equivalent variance in dominance effects of single substitutions (i.e., the *d* increments of the random walk; Equations 4 and 28). As with the additive model, Equations 6‐7 predict a transient increase in F1 fitness in the early stages of divergence, as long as the second terms remain small. But as the number of substitutions (*d*) increases, the second terms start to grow, causing a steady fitness decline (ultimately giving rise to the pattern shown in Fig. 1b). To illustrate this, we repeated our simulations after allowing for variable phenotypic dominance among the new mutations (see Appendix 2 for full details). The results, shown as dark red curves in Figure 2c, show a clear pattern of transient heterosis—optimal outbreeding—followed by a linear decline in log fitness: the F1 clock (see row 3 Table 1). Note that heterosis is greater with phenotypic dominance (compare peaks of yellow and dark red curve in Fig. 2c) because more highly recessive deleterious mutations now segregate (i.e. mutations that are also phenotypically recessive).

We can also think of this result as exemplifying two regimes. In the early stages of divergence, the phenotypic distance between the parents increases faster than the cloud of possible F1. This means that most F1 trait values will be intermediate between the parental values. Later, the parents stop diverging phenotypically (because of effective stabilizing selection acting on their traits), but the cloud of possible F1 continues to expand. This leads to transgressive trait variation, with the F1 values lying outside of the range of the parental values. It is notable that both of these regimes have been observed in quantitative traits from F1 hybrids: transgressive variation sometimes decreases with *d*, and sometimes increases with *d* (Stelkens & Seehausen, 2009, ; see also Appendix 1 and Figure S2 for more details).

#### The tick rate of the F1 clock

Equation 7 gives the tick rate of the F1 clock as $nV_{a\delta }$, which is also the rate of expansion of the cloud of possible F1 phenotypes. This quantity summarizes the distribution of factors fixed, which, as previous work has shown, will depend on many different aspects of the system's biology (e.g. Charlesworth, 1992; Griswold, 2006; Matuszewski et al., 2014; Orr, 1998; Schneemann et al., 2020; Yamaguchi & Otto, 2020; Yeaman & Whitlock, 2011).

**Figure 3:**
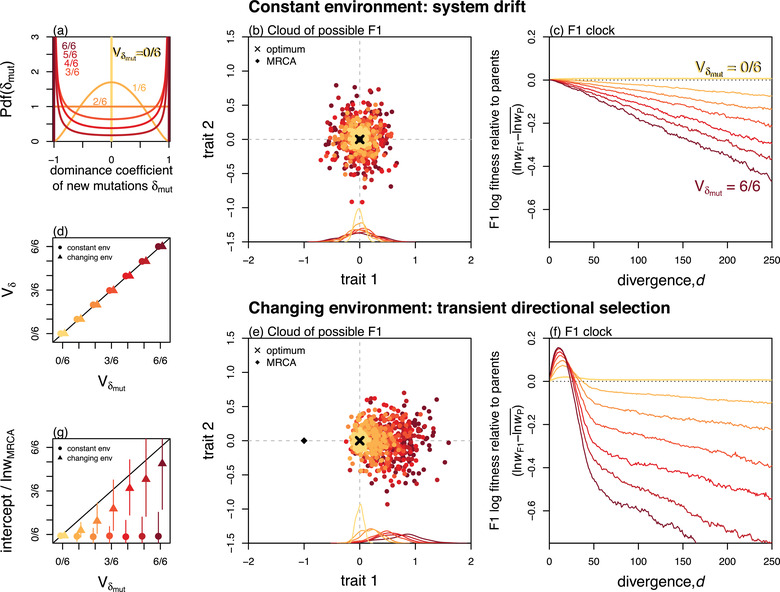
Deleterious effects of phenotypic dominance depend on the distribution of new mutations and the history of directional selection. **(a)** The distribution of dominance coefficients for new mutations used in each set of simulations. These were 7 different beta distributions, with vanishing means and 7 different variances, ranging from additivity (yellow: $V_{\delta_{\mathrm{mut}}}=0/6=0$) to complete dominance or recessivity (dark red: $V_{\delta_{\mathrm{mut}}}=6/6=1$). **(b)**–**(c)** Simulations under constant environmental conditions show that **(b)** the variance in the cloud of possible F1s, and **(c)** the tick rate of the F1 clock both increase steadily with $V_{\delta_{\mathrm{mut}}}$. **(d)** This is because, for these simulations, the dominance coefficients of fixed mutations reflect those of the new mutations, such that $V_{\delta }\approx V_{\delta_{\mathrm{mut}}}$. **(e)** Directional selection can lead to directional dominance, causing the cloud of possible F1s to “overshoot” the optimum in the direction of the past adaptation. **(f)** Directional dominance creates transient heterosis as the parental lines evolve toward the new optimum, but a deleterious overshoot once the new optimum is reached. **(g)** The result is that, after an initial period, the F1 clock ticks at the same rate, but with an intercept which also increases with $V_{\delta_{\mathrm{mut}}}$. (b) and (e) show 100 simulations stopped after d=50 substitutions, and with parameters chosen to best visualise the clouds (N=100, n=2). (c)–(d) and (f)–(g) show means across 100 replicate simulations with n=20 and N=1000 to generate a steady clock. All panels use $U=\bar{s}=0.01$.

One important determinant of the tick rate is likely to be the distribution of dominance coefficients of new mutations. In Figure 2c, we used an empirically motivated model, in which larger‐effect mutations tended to have extreme levels of dominance (see Appendix 2; Orr, 1991; Manna et al., 2011), however, the effects of mutational dominance are easier to see with a simpler model, where they can vary independently of the additive effects. As such, we repeated our simulations using the model of Manna et al. (2011), where the dominance coefficient of each new mutation on each trait was drawn from a shifted beta distribution, with a vanishing mean (such that mutations were semi‐dominant on average) and variance $V_{\delta_{\mathrm{mut}}}$. We simulated under seven different variances, including the extremes of $V_{\delta_{\mathrm{mut}}}=0$ (phenotypic additivity) and $V_{\delta_{\mathrm{mut}}}=1$, such that new mutations were either fully dominant or fully recessive with a 50:50 probability. The seven distributions we used are illustrated in Figure 3a.

Figure 3b shows clouds of F1 phenotypes for sets of 100 replicate simulations, each with n=2 for visualization. In all cases, the cloud of possible F1 is centred on the optimum, but grows in width with $V_{\delta_{\mathrm{mut}}}$. Figure 3c shows the consequence of this F1 cloud expansion for the F1 clock. Here, to match Fig. 2c, we simulated with n=20 traits, but with a larger population size, so that parental populations remained well adapted and thus had little potential for F1 heterosis (see Figure S3 for other parameter regimes). While this may not hold under all possible regimes, all our simulation results show that the F1 tick rate increases steadily with the variance in mutational dominance, $V_{\delta_{\mathrm{mut}}}$. This is because the heterozygous effects of the substitutions become more variable with $V_{\delta_{\mathrm{mut}}}$. To see this, we can isolate the contribution of dominance to $V_{a\delta }$ that will be captured by the key quantity $V_{\delta }$, defined as

(8) $$\begin{array}{c} V_{\delta }\equiv V_{a\delta }/V_{a} \end{array}$$

where $V_{a}$ is the variance of the fixed additive effects, averaged over traits (Equation 29), and $V_{\delta }$ captures the variability in dominance coefficients of fixed effects, as well as any potential correlations between fixed additive effects and their associated dominance coefficient (see section 1.1.1 of Appendix 1). Figure 3d (circles) shows that for these simulations the quantity $V_{\delta }$ is very close in value to the mutational variance in dominance coefficients, i.e. that $V_{\delta }\approx V_{\delta_{\mathrm{mut}}}$ (see also Schneemann et al. 2020).

#### Directional selection and directional dominance

Results above assume that dominance deviations have no tendency to point in any phenotypic direction, such that the cloud of possible F1s is centered on the midparent, and all $E(\Delta_{i})=0$ (see Fig. 2b). However, it is well known that directional selection can lead to directional dominance, as with Haldane's Sieve (Billiard et al., 2021; Crnokrak & Roff, 1995; Frankham, 1990; Haldane, 1924, 1927), especially if adaptation is from new mutations (Orr & Betancourt, 2001). This is because populations evolving in a particular direction preferentially fix alleles that are dominant in that direction.

To understand the effects of directional dominance for F1 fitness, let us consider the most extreme case, where both populations adapt independently to identical environmental change, so that all of the dominance deviations point in the same direction. In this case, the cloud of possible F1 becomes shifted in the direction of the past evolutionary change. This is illustrated in Figure 3e, where a bout of directional selection on trait 1 leads to $E(\Delta_{1})>0$. To understand why this happens, let us consider the evolution of trait 1 during the bout of adaptation. If we denote the F1 trait as *z*_F1, 1_, and the midparental value as *z*_MP, 1_, then Equations 2 and 5 imply that:

(9) $$\begin{array}{cc} E\left({\left(z_{F1,1}-o_{1}\right)}^{2}\right) & ={\left(z_{\mathrm{MP},1}-o_{1}\right)}^{2}+E\left(\Delta_{1}^{2}\right)-2\left|\left(z_{\mathrm{MP},1}-o_{1}\right)\right|E\left(\Delta_{1}\right) \end{array}$$

(10) $$\begin{array}{cc} & =V_{\Delta_{1}}+E^{2}\left(\Delta_{1}\right),\mathrm{if}z_{\mathrm{MP},1}=o_{1} \end{array}$$

The important point about Equation 9 is that it contains both positive and negative terms. This is because, during the adaptation phase, the directional dominance can increase F1 fitness, by taking its trait value closer to the still‐distant optimum. But as shown by Equation 10, once the new optimum is approached, the directional dominance leads to a permanent and deleterious “overshoot” of this optimum (see also Figure S4, and Ono et al. (2017) for a related phenomenon shown in yeast).

If the adaptation to the new optimum required *d*_dir_ substitutions, the expected log fitness of the F1 after this period (i.e., once system drift at the new optimum has begun) is:

(11) $$\begin{array}{cc} E\left(\ln w_{F1}\right) & \approx \ln w_{\mathrm{MRCA}}V_{\delta_{\mathrm{dir}}}-\left(d-d_{\mathrm{dir}}\right)nV_{a}V_{\delta },\;\mathrm{if}\;z_{\mathrm{MP}}=o,d>d_{\mathrm{dir}} \end{array}$$

where $V_{\delta_{\mathrm{dir}}}$ is the variance in dominance coefficients of the adaptive substitutions, and $\ln w_{\mathrm{MRCA}}$ is the log fitness of the maladapted ancestor. Comparing eq. 11 to earlier results (Equations 6 and 7), we see that the overshoot adds a new term. The history of directional selection leads to a non‐zero intercept for the F1 clock.

Complete simulations of this scenario are reported in Figure 3f. Comparison of Figure 3f and 3c shows clearly the transient increase in F1 fitness (Equation 9), and the deleterious overshoot once the new optimum is approached (Equation 10). After the initial period, the F1 clock continues to tick at the same rate as before (see triangles in Figure 3d), but with a permanent intercept (Equation 11). Fitting linear regressions to the F1 clocks in Figure 3f after excluding the first 50 substitutions allowed us to calculate this intercept. Figure 3g (triangles) confirms that the intercept is also affected by the input of new mutations, such that $V_{\delta_{\mathrm{dir}}}\propto V_{\delta_{\mathrm{mut}}}$.

Results above concern directional selection in a common direction, but they generalize readily. For example, if each parental population underwent directional adaptive change on a different phenotypic trait, the resulting directional dominance would lead to an F1 with a mixture of the derived traits of the two parental lines. This is described as “trait mismatch” by Thompson et al. (2021). In all cases, with variable phenotypic dominance, a history of past directional selection can lead to an additional loss of fitness for the F1.

### THE LUCKY BENEFICIAL EFFECTS OF DOMINANCE

So far, we have considered the typical effects of dominance in the F1, by averaging over the possible evolutionary histories. These effects are generally deleterious, and any heterosis tends to be transient. Nevertheless, even when effects are deleterious on average, by chance alone, some realisations of the evolutionary process will take the F1 closer to the optimum. In Fisher's geometric model, these “lucky” outcomes are far more likely when the number of phenotypic traits, *n*, is small. This is because random changes are more likely to go in the right direction when the dimensionality of the landscape is low (Fisher, 1930; Orr, 2000).

To see this, let us first consider again the scenario illustrated in Figures 2c and 3b‐c, where parental populations remain well adapted to a single fixed optimum. We assume that the $\Delta_{i}$ have vanishing means (i.e., no directional dominance), and are approximately normally distributed. The normality is justified by the central‐limit‐like behaviour arising from the sum in eq. 4. With these assumptions, the coefficient of variation in log F1 fitness is approximately

(12) $$\begin{array}{cc} \mathrm{CV}\left(\ln w_{F1}\right) & \equiv \frac{\sqrt{\mathrm{Var}\left(\ln w_{F1}\right)}}{|E\left(\ln w_{F1}\right)|}\\ & \approx \sqrt{\frac{2}{n}},d\gg 1 \end{array}$$

Equation 12 implies that, when *n* is large, the F1 clock will tick in a relatively deterministic way. When *n* is small, by contrast, increasing the divergence between the parental lines may lead to the chance re‐appearance of heterosis, even after many generations of low fitness F1. This is confirmed by simulations reported in Figure S5a‐b.

#### Lucky beneficial effects in a novel environment

**Figure 4:**
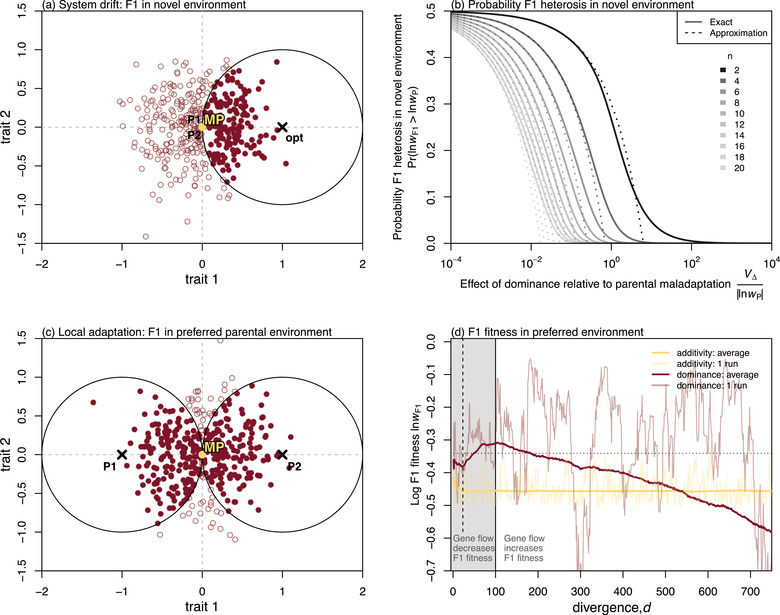
“Lucky” beneficial effects of dominance in heterogeneous environments. **(a)** Even when two parental lines are well adapted to similar environments, their F1 may be well adapted to a novel environment, simply by chance. These fitter F1 (solid dark red points) lie within the circle shown, with the new optimum at its centre. **(b)** The probability of such fitter F1 appearing decreases rapidly with *n* (the dimensionality) and with $V_{\Delta }/\left|\ln w_{P}\right|$ (the size of the dominance deviations relative to the maladaptation of the parents). The approximation is eq. 13 and the exact result is Equation 44. **(c)** When the parents are locally adapted to two different environments, dominance might yield an F1 that is much fitter in one of the two environments, potentially leading to asymmetrical gene flow. **(d)** Simulations of the F1 of locally adapted parents. F1 were scored in the “preferred” parental habitat, i.e. the habitat to which they were better adapted. Simulations began with the MRCA intermediate between the two optima, and the parents adapted to their optima rapidly, always before d=25 (see vertical dotted line). With additivity, F1 fitness remained roughly constant thereafter (yellow curves), but with dominance, expected F1 fitness continued to increase with divergence (dark red curves), until a maximum roughly predicted by eq. 15 (horizontal dotted line). However, changes in F1 fitness were erratic for any single run (see fainter curves). The result is that gene flow, which reduces *d*, can either increase or decrease F1 fitness with dominance. Simulations in (d) were the same as in Fig. 3e, with the extremes of $V_{\delta_{\mathrm{mut}}}=0$ and 1, and averages taken over all possible pairs of the 200 parental populations; see also Figure S7.

The lucky beneficial effects of dominance may be particularly consequential when hybrids are formed in novel environments, to which one or both parental lines are severely maladapted. In the additive model, F1 heterosis can only appear when the parents are maladapted in different ways (Schneemann et al., 2020; Simon et al., 2018; Yamaguchi & Otto, 2020). For example, hybrids between parents adapted to low and high altitudes might thrive at intermediate altitudes (Wang et al., 1997). With phenotypic dominance, however, hybrid advantage might appear under a broader range of conditions (row 1 Table 1). Consider, for example, the situation shown in Figure 4a, where two genomically divergent, but phenotypically similar parental lines hybridize in a novel habitat. In this case, the midparent matches the parental phenotypes ($\ln w_{\mathrm{MP}}\approx \ln w_{P}$), and so the effects of dominance will be deleterious on average (Equation 6). Nevertheless, for some divergence histories, the F1 will be fitter by chance. The probability of this lucky heterosis can be derived by noting that Equation 5 has a non‐central chi‐squared distribution if the $\Delta_{i}$ are normal. The probability therefore depends on *n* and the ratio $V_{\Delta }/\ln w_{P}$, which compares the sizes of the dominance deviations to the maladaptation of the parents. Figure 4b plots this probability for a range of values, and we also have the approximation:

(13) $$\mathrm{Pr}\left(\ln w_{F1}>\ln w_{P}\right)\approx \frac{1}{2}\left(1-\frac{\left(n-1\right)}{\sqrt{2\pi }}\sqrt{\frac{V_{\Delta }}{|\ln w_{P}|}}\right)$$

(see Appendix 1). As shown in Figure 4b, the maximum probability of heterosis is $\frac{1}{2}$, and it applies when dominance effects are small compared to the maladaptation of the parents such that $V_{\Delta }\ll \left|\ln w_{P}\right|$ (toward the left of Fig. 4b). This implies that any fitness gain due to heterosis would also be very small, and simply reproduces Fisher's result that very small changes have a 50% chance of being beneficial (Fisher, 1930). Conversely when the dominance effects are very large, such that $V_{\Delta }\gg \left|\ln w_{P}\right|$ (toward the right of Fig. 4b), the F1 are almost certain to overshoot the new optimum. Therefore, the area of interest concerns $V_{\Delta }\approx \left|\ln w_{P}\right|$ (toward the centre of Fig. 4b), and in this regime, the probability of heterosis declines rapidly with *n* (see also Fig. S6). Only when *n* is small is there a non‐negligible chance that dominance effects are both substantial and beneficial.

#### Hybrids between locally adapted parents

Now let us consider the situation shown in Figure 4c. This panel represents the outcome of divergent selection (Schluter, 2000), where each parental line is well adapted to a different habitat, characterized by different phenotypic optima. In this case, the midparent lies midway between the optima, such that, in either habitat $\ln w_{\mathrm{MP}}\approx \ln w_{P}/4$, with $\ln w_{P}$ denoting the fitness of the maladapted parental line. In this scenario, results in Figure 4b now describe the probability of the F1 being fitter than the midparent in one of the two parental habitats. In most cases, the F1 will be fitter in one of the habitats than the other, and in this “preferred habitat”, its expected log fitness is:

(14) $$\begin{array}{cc} E\left(\ln w_{F1_{\mathrm{pref}}}\right) & =\frac{\ln w_{P}}{4}-nV_{\Delta }+\sqrt{\frac{2V_{\Delta }\left|\ln w_{P}\right|}{\pi }} \end{array}$$

Equation 14 neatly separates the negative and positive effects of dominance in its second and third terms, and shows that the deleterious effects—but not the beneficial effects—grow with *n*. Equation 14 also shows that the fitness benefits of dominance will be greatest at intermediate values of $V_{\Delta }$, and therefore, at intermediate levels of divergence (Equations 6‐7). This means that, in contrast to the scenario shown in Figure 3e–f, F1 fitness in one of the two habitats, might continue to increase even after two parental populations have adapted to their new optima. At this intermediate level of divergence, when the fitness benefit is greatest, we find:

(15) $$\begin{array}{cc} \max\left(E\left(\ln w_{F1_{\mathrm{pref}}}\right)\right) & =\ln w_{\mathrm{MP}}\left(1-\frac{2}{\pi n}\right), \end{array}$$

so, at suitable levels of divergence, phenotypic dominance can yield a substantial fitness increase for the F1 over the midparent. However, these lucky effects of dominance occur only in one of the two habitats and only when *n* is small. All of these results are confirmed in Figure 4d, which shows simulation results with n=2 (see also Figure S7). Results confirm that on average (solid lines), F1 fitness with dominance initially increases with *d*, before reaching a maximum approximated by Equation 15, and then decreasing. However, Equation 12 still applies, and so for any single realization of the divergence history (faint lines in Figure 4d) the fitness of F1 with phenotypic dominance is highly erratic.

Taken together, these results imply that phenotypic dominance can cause an asymmetry in F1 fitness between the two parental habitats (see row 4 Table 1, and top row Fig. S8). This is a form of “dominance drive” (Barton, 1992; Mallet & Barton, 1989), and could lead to asymmetrical gene flow between locally adapted populations after secondary contact. The results also imply that the gene flow, which by definition reduces *d*, may have an unpredictable effect on F1 fitness. In some cases, homogenisation of the parental genomes will increase the fitness of their F1, but in other cases, it will lead to a switch in the direction of the gene flow (as the F1 becomes adapted to the other parental habitat), or even to a substantially lower F1 fitness. In the latter case, the outcome would resemble reinforcement selection, albeit via a completely different route.

### THE INTERACTION OF PHENOTYPIC DOMINANCE WITH UNIPARENTAL INHERITANCE

**Figure 5:**
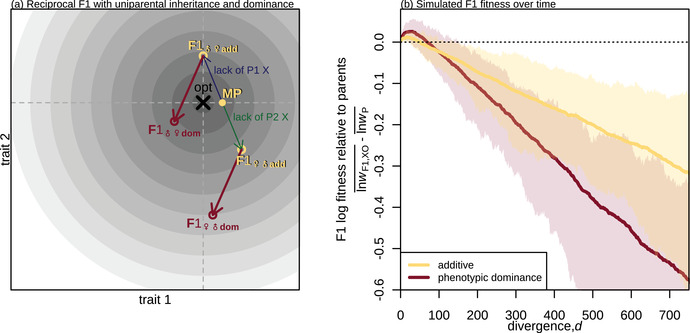
Uniparental effects interact with dominance to determine F1 fitness. Both phenotypic dominance and uniparental inheritance/expression cause the F1 to deviate from the midparent (MP). **(a)** An illustration of heterogametic (XO) offspring, from a reciprocal F1 cross: i.e., including both the male‐female and female‐male cross directions of the same parental lines. Yellow points show the reciprocal F1 under additivity (F1_♀♂,add_, F1_♂♀,add_) and dark red points show the reciprocal F1 with phenotypic dominance (F1_♀♂,dom_, F1_♂♀,dom_). The dark blue and green lines indicate the deviations due to the absent paternal X, and are equal and opposite for the two cross directions. The dark red lines indicate the dominance deviations due to heterozygosity on the autosomes, and are identical for both cross directions. Both sets of deviations are expected to grow with the divergence, *d*. **(b)** Simulation results of log fitness in XO F1 hybrids, averaged across the two cross directions. Results show that the F1 clock appears under additivity with uniparental effects. Plotting conventions and simulation runs are identical to those shown in Figure 2c, except that a proportion x=1/4 of the divergent sites were randomly assigned to the X‐chromosome before forming the hybrids. As a result, optimal outbreeding and the F1 clock appear not only with dominance (dark red curves) but also with additivity (yellow curves).

Let us now expand the model to include the uniparental inheritance or expression of some of the divergent alleles, for example on sex chromosomes or mitochondrial genomes (Fraïsse et al., 2016; Simon et al., 2018). Like phenotypic dominance, uniparental inheritance leads to unpredictable deviations in hybrid phenotype. This is because the complete set of additive effects may be co‐adapted in the parental lines, for example, due to compensatory changes. But when hybrids inherit some parts of their genome from only one parent, they may lack some co‐adapted alleles from the other parent. The one key difference from dominance is that, under uniparental inheritance, the direction of the deviation from the midparent is opposite for the two cross directions. This is illustrated in Figure 5a.

In this figure, and throughout this section, we assume a concrete example of uniparental effects, although results generalize easily to other cases. Our example involves sex chromosomes, where the heterogametic sex are effectively XO (i.e. where males carry only the maternal X, and the Y is either missing or highly degenerate). We further assume a common form of dosage compensation where X‐linked alleles have identical effects in homozygous and hemizygous state, as found in e.g. *Diptera* and *Hemiptera* (Deng et al., 2011; Gu & Walters, 2017; Mank et al., 2011). This last assumption ensures that non‐hybrid offspring of both sexes are phenotypically identical (i.e., that male and female offspring from a P1 × P1 cross are equally fit). It also means the homogametic sex still experience dominance effects at X‐linked loci, such that we can study the interaction between uniparental and dominance effects. The consequences of some alternative forms of dosage compensation are addressed in Fraïsse et al. (2016), and will be discussed briefly below.

In the case described, the absence of the paternal X causes the heterogametic F1 hybrid to deviate from the midparent, even under phenotypic additivity. These deviations are equal and opposite for the two cross directions (see the blue and green arrows in Fig. 5a). If there is phenotypic dominance for alleles on the autosomes, then this leads to further deviations that apply identically to both cross directions (see the dark red arrows in Fig. 5a). In the sections below, we show how uniparental effects and phenotypic dominance combine to determine F1 fitness.

#### The F1 clock under dominance and uniparental inheritance

If we consider any single F1, then uniparental effects and phenotypic dominance have essentially the same consequences. As such, they represent alternative, but non‐exclusive explanations of the F1 clock and optimal outbreeding (row 3 Table 1). To see this, let us consider the expected log fitness of an XO F1 hybrid. If we assume that a fraction, *x*, of the *d* divergent alleles are X‐linked, then from eq. 5 and published results (Fraïsse et al., 2016; Simon et al., 2018), we find:

(16) $$\begin{array}{cc} E\left(\ln w_{F1,\mathrm{XO}}\right) & =\frac{\bar{\ln w_{P}}}{2}\left(1+x^{2}\right)-dnV_{a}V_{\delta }\left(1-x\right)-dnV_{a}x\left(1-x\right) \end{array}$$

Equation 16 still predicts optimal outbreeding, but there are now two terms that reduce F1 fitness. The second term captures the dominance deviations from the autosomes, and the third term captures the lack of co‐adaptation between paternal autosomes and the absent paternal X.

Figure 5b illustrates these results. We returned to our simulated data, and formed heterogametic F1 in the way described. Results show that optimal outbreeding and the F1 clock now appear under the additive model too (yellow curves; Fraïsse et al., 2016; see also Fig. S5c‐d). Phenotypic dominance simply accelerates the fitness decline (dark red curves).

#### Haldane's rule

If we consider fitness differences between heterogametic and homogametic hybrids, then the uniparental effects and phenotypic dominance tend to push in different directions. If the parents are well adapted, then the additive model predicts lower fitness for the heterogametic sex, in accord with Haldane's Rule (row 5 Table 1; Haldane, 1922; Barton, 2001; Fraïsse et al., 2016; Simon et al., 2018). This is due to the loss of co‐adaptation, described above (see the third term of eq. 16). However, phenotypic dominance has the opposite effect, because it tends to makes heterozygosity deleterious, and heterogametic F1, being hemizygous for the X, lack the deleterious heterozygosity on the X (to see this, compare the second terms of eqs. 7 and 16).

To see how these two effects balance, let us consider the difference in log fitness between heterogametic and homogametic F1. Using Equations (7), (8), and 16, we find:

(17) $$\begin{array}{cc} E\left(\ln w_{F1,\mathrm{XO}}-\ln w_{F1,\mathrm{XX}}\right) & =\frac{\bar{\ln w_{P}}}{2}x^{2}-dnxV_{a}\left(1-x-V_{\delta }\right) \end{array}$$

Haldane's Rule holds on average if eq. 17 is negative. If parents are well adapted, or divergence is substantial, then the second term in Equation 17 dominates and we expect Haldane's Rule on the condition that

(18) $$V_{\delta }<\left(1-x\right).$$

**Figure 6:**
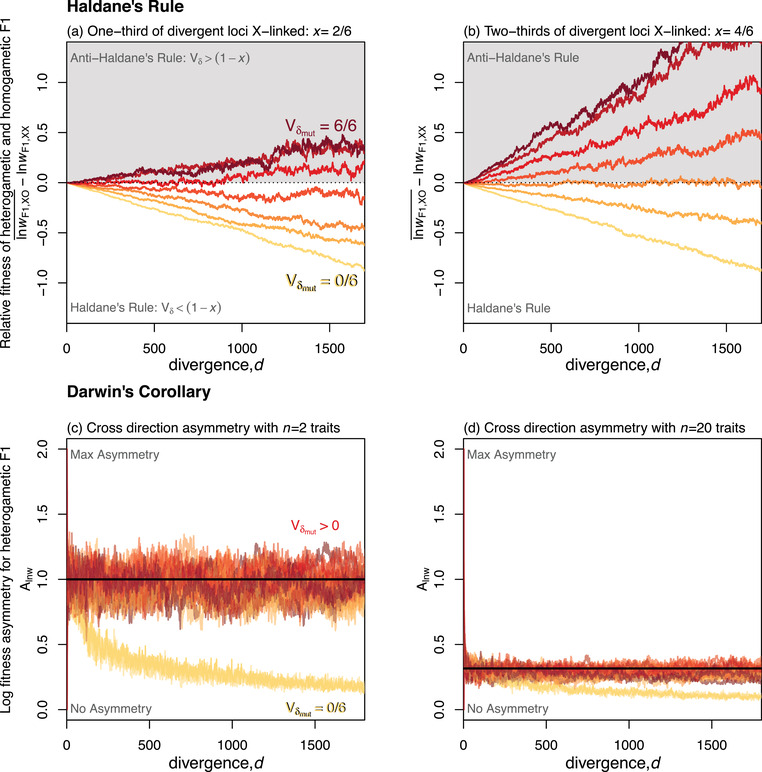
Differences between F1 cross types with uniparental inheritance and variable phenotypic dominance. **(a)–(b)** Phenotypic dominance can lead to violations of Haldane's Rule. Curves show the difference in mean log fitness between heterogametic and homogametic F1, at various levels of parental divergence. Curves below the horizontal dotted line indicate that heterogametic F1 are less fit, such that Haldane's Rule holds. Colours correspond to those in Figure 3, and show the same 7 values of $V_{\delta_{\mathrm{mut}}}$. Haldane's Rule always holds with additivity (yellow lines; $V_{\delta_{\mathrm{mut}}}=0$), but can be violated if dominance coefficients are highly variable (darker colours), or if the X chromosome is very large—compare (a) to (b). **(c)–(d)** Phenotypic dominance enhances log fitness asymmetry between reciprocal F1. Curves show the asymmetry measure of Equation 19, measured for XO hybrid offspring from the two cross directions. Results under additivity (yellow), differ qualitatively from those with phenotypic dominance (all other colours), and the latter all show approximately the same result: $A_{\ln w}\approx \sqrt{\frac{2}{n}}$ (black horizontal lines). Plots include 28 sets of simulations, using two populations sizes: N=100,1000; two sizes of X chromosome: $x=\frac{1}{3},\frac{2}{3}$; and the 7 $V_{\delta_{\mathrm{mut}}}$ values shown in Fig. 3a. Each curve represents the mean across 100 simulation replicates, all with $U=\bar{s}=0.01$; (a)–(b) used n=20 and N=100. Note that the divergence was simulated under strictly biparental inheritance, to ensure that $V_{\delta }\approx V_{\delta_{\mathrm{mut}}}$.

Equation 18 confirms that Haldane's Rule will always hold under additivity (when $V_{\delta }=0$), but that variable phenotypic dominance can lead to violations (i.e., ‘anti‐Haldane's Rule'), especially when the dominance coefficients are highly variable, or if the X is very large (rows 5–6 Table 1). This is confirmed by the simulations shown in Figure 6a–b. These plots use the simulation runs from Fig. 3b, for which the dominance coefficients match the mutational input ($V_{\delta }\approx V_{\delta_{\mathrm{mut}}}$). The results show that Haldane's Rule is indeed violated when $V_{\delta_{\mathrm{mut}}}>\left(1-x\right)$.

#### Darwin's corollary

Let us now consider the fitness differences between the F1 cross directions, that is a cross where P1 is dam (P1♀× P2♂) compared to a cross where P1 is sire (P2♀× P1♂). In stark contrast to Haldane's Rule, phenotypic dominance works together with uniparental effects to generate this pattern. To see this, we will use the following measure of log fitness asymmetry, where the absolute difference between the cross directions is normalized by the absolute mean:

(19) $$A_{\ln w}\equiv \frac{\left|\ln w_{F1,}-\ln w_{F1,}\right|}{\frac{1}{2}\left|\ln w_{F1,}+\ln w_{F1,}\right|}$$

Here, ♀♂ and ♂♀ denote the two cross directions, and the statistic is bounded at $0\leq A_{\ln w}\leq 2$.

Fraïsse et al. (2016) studied F1 asymmetry under the additive version of Fisher's model, and showed that asymmetry could appear only if the midparental phenotype was maladapted. The reason is clear from Figure 5a. When the midparent matches the optimum, the equal and opposite deviations (blue and green arrows), will lead to identical fitness loss.

Furthermore, even if the midparent is maladapted, its distance from the optimum must be large relative to the deviations for substantial asymmetry to appear. But in a stable environment, the parents (and therefore the midparent) remain close the optimum, while the deviations grow with *d* (e.g. eq. 7). As such, the F1 in both cross directions are expected to become less fit, and the difference between their fitnesses is expected to become smaller. This implies that fitness asymmetry between the cross directions will decline with *d* under the additive model. This is confirmed by the yellow curves in Figure 6c–d.

Adding phenotypic dominance to the model qualitatively changes this result. Because the dominance deviations also grow with *d*, levels of asymmetry can remain large, even at high divergence (row 7 Table 1). An illustrative case is shown in Figure 5a. Moreover, with dominance, we find that to a rough approximation:

(20) $$E\left(A_{\ln w}\right)\approx \sqrt{\frac{2}{n}},\mathrm{if}V_{\delta }>0$$

so the normalized asymmetry in log fitness is predicted to remain roughly constant at all levels of divergence, and to depend solely on the number of traits, *n*. This is confirmed by the simulations with dominance shown in Figure 6c–d. For all of the parameter values we simulated, as long as there was phenotypic dominance ($V_{\delta_{\mathrm{mut}}}>0$), then asymmetry levels remained roughly constant, and were close to the prediction of eq. 20 (see also Figure S5e– f).

The arguments in this section show that under Fisher's geometric model, Darwin's Corollary can be explained as another “lucky” beneficial effect of dominance, where the good luck for one cross direction is balanced by bad luck for the other. The consequence is that – with phenotypic dominance, but not with additivity – Darwin's Corollary is predicted at high levels of genomic divergence (row 7 Table 1).

## Discussion

This paper has investigated the effects – both negative and positive – of variable phenotypic dominance on F1 hybrid fitness. We have shown that these effects are often governed by a quantity $V_{\delta }$, which captures the variability in the dominance coefficients of the fixed differences between hybridizing populations, but with the variability defined across realisations of the divergence history (see eq. 8, and section 1.1.1 in Appendix 1 for a formal definition). As a result, the importance of phenotypic dominance to F1 hybrid fitness will usually depend on the typical size of $V_{\delta }$ in nature.

### Can we measure $V_{\delta }$ directly?

In principle, $V_{\delta }$ might be estimated using a quantitative genetics approach (Clo et al., 2021; Hill, 1982; Lynch, 1991), either applied to fitness (De Sanctis et al., 2022; Schneemann et al., 2020) or to the underlying traits, if their relationships to fitness are known (though see Martin, 2014). However, as $V_{\delta }$ describes the variance across replicates of the divergence process, its estimation would require very large amounts of data.

Another, indirect approach to estimating $V_{\delta }$ uses the properties of new mutations. We have shown that, in some cases, $V_{\delta }$ reflects the mutational input, such that $V_{\delta }\approx V_{\delta_{\mathrm{mut}}}$ (Fig. 3d). Manna et al. (2011) showed that the regression slope of homozygous and heterozygous selection coefficients carries information about $V_{\delta_{\mathrm{mut}}}$ (see their eq. 13). Their re‐analysis of several mutation accumulation experiments estimated this slope to be on average 0.27 (see also Charlesworth & Charlesworth, 1998; Lynch & Walsh, 1998; Szafraniec et al., 2003), which corresponds to ${\hat{V}}_{\delta_{\mathrm{mut}}}=0.08$. However, the same slope could also arise without phenotypic dominance ($V_{\delta_{\mathrm{mut}}}=0$) if the wildtypes were maladapted.

While $V_{\delta }$ therefore appears very difficult to estimate, existing data on F1 hybrid fitness might nonetheless provide bounds on its typical value.

### What data might place a lower bound on $V_{\delta }$?

One of the goals of this work, as summarized in Table 1, was to identify patterns in the F1 fitness data that only dominance might generate. Observing these patterns would thereby demonstrate that $V_{\delta }$ must be greater than zero. However, such patterns were surprisingly hard to find. This is mainly because the effects of phenotypic dominance so often resemble the effects of uniparental inheritance or expression—with both acting to displace the F1 phenotype from the midparent in an unpredictable direction (Fig. 5b,c; Fraïsse et al., 2016). Moreover, the ubiquity of such uniparental effects is evident in the ubiquity of Darwin's Corollary—fitness differences between the cross directions (Fig. 1d; Kölreuters, 1766; Darwin, 1859, Ch. 8; Turelli & Moyle, 2007; Muller, 1942).

Nevertheless, as summarized in Table 1, there are several patterns in the data that do imply a role for phenotypic dominance. These patterns include (i) F1 heterosis for the reciprocal F1 of phenotypically identical parents (Fig. 4a,b); (ii) reciprocal F1 between locally adapted parents being much fitter in one of the two parental environments (Fig. 4c); (iii) violations of Haldane's Rule due to deleterious heterozygosity (Fig. 6a,b); and (iv) the appearance of Darwin's Corollary at very large genetic distances (Fig. 6c,d).

Unfortunately, in each case, there are complications that make it difficult to conclude that the observations really are due to phenotypic dominance. For example, regarding (i), it is very difficult to know that parental lines are truly identical for all of the relevant traits (Martin, 2014); regarding (ii) parental habitats may be under different forms or strengths of selection, such that phenotypically intermediate F1 are nevertheless much fitter in one environment (see Fig. S8); regarding (iii) violations of Haldane's Rule may appear for other reasons, notably silencing of sex chromosomes in the homogametic sex (Fraïsse et al., 2016), and this does seem plausible in the best‐studied examples (*Teleogryllus* crickets: Simon et al., 2018; Moran et al., 2017; but see also Rayner et al., 2021; marsupial mammals: Watson & Demuth, 2012; *Drosophila*: Sawamura, 1996; and *Xenopus*: Malone et al., 2007); finally, regarding (iv) levels of fitness asymmetry can vary erratically with genetic distance (see Fig. S5e,f), and moreover the relevant measure of asymmetry (Equation 19), can be difficult to obtain at low divergences—where parental fitness cannot serve as a proxy for the optimal fitness—and at very high divergences, where zeros cannot be log transformed.

As a result of these complications, our work has identified only one pattern in F1 fitness data from which the action of phenotypic dominance can be confidently inferred. This pattern—described in row 8 of Table 1—is a particular form of environment‐dependent heterosis, where both F1 cross directions outperform both parents in one environment (as in Fig. 1a), while both parents outperform both F1 in a second environment (as in Fig. 1b). (The second environment might involve the presence of novel stressors, to which the parental lines are not normally exposed). The F1 heterosis in the novel environment would appear either as a “lucky” effect (Figs. 4a,b; Fig. S7), or as a predictable consequence of Haldane's Sieve and past adaptation (Fig. 3e,f). How common, then, is this pattern? While there are many excellent examples of environment‐dependent heterosis, they rarely match the pattern described exactly—either because F1 fitness is intermediate in one of the environments (e.g., Crespel et al., 2012, 2012; Martins et al., 2019; Munaro et al., 2011; Vetukhiv & Beardmore, 1959; Walter et al., 2020; Wu & Campbell, 2006; Wang et al., 2022), or because F1 fitness was not available for the individual cross directions (e.g., Benyi & Gall, 1981; Dittrich‐Reed, 2013; Domgínguez & Albornoz, 1987; Harrison, 1962; Maynard Smith, 1956; Stojanova et al., 2021; Wagner et al., 2021). As such we have found only two possible examples of the complete pattern. The first, from Pereira et al. (2014), shows temperature‐dependent F1 heterosis in the copepod *Tigriopus californicus*; the second, from Hahn and Rieseberg (2017), shows drought‐dependent F1 heterosis between particular populations of common ragweed *Ambrosia artemisiifolia*. The relevant data from these studies are reproduced in Figure S9. While there are caveats in each case (slightly different measures of fitness or large confidence intervals) data of this kind are the strongest evidence for the importance of phenotypic dominance to F1 fitness.

### What data might place upper bounds on $V_{\delta }$?

In contrast to the previous section, data placing upper bounds on $V_{\delta }$ in nature are relatively easy to find. These data suggest that dominance effects—even if they are present—cannot be very large.

The first relevant observation is the paucity of exceptions to Haldane's Rule – especially once we exclude taxa with X or Z silencing in the homogametic sex (Haldane, 1922; Coyne & Orr, 2004, Ch. 8; Schilthuizen et al., 2011). We have shown that Haldane's Rule is predicted only when $V_{\delta }<1-x$, where *x* is the proportion of divergent loci that are X‐linked (eq. 18; Fig. 6a‐b). Moreover, the $V_{\delta }$ in this equation applies to dominance effects at X‐linked loci, which are predicted to be more variable than those on the autosomes (Charlesworth et al., 1987), suggesting a tighter upper bound for genome‐wide $V_{\delta }$. In addition, data from multiple *Drosophila* species show an increase in the strength of Haldane's rule with *x* (Turelli & Begun, 1997). It follows from Equation 17, that such an increase is predicted only on the condition that $V_{\delta }<1-2\max\left(x\right)$, further tightening the bound.

The second observation that provides an upper bound for $V_{\delta }$ is hybrid breakdown—the fact that later‐generation hybrids are very often less fit than the initial F1 (Fraïsse et al., 2016; Muller, 1940; Vetukhiv, 1954, Table S1). This is attested both by experimental crosses, and by the relative absence of recombinant hybrids in wild tension zones that are dominated by viable and fertile F1 (e.g., Milne, 2019). An explanation for this hybrid breakdown is that later‐generation hybrids (just like recombinant haploid hybrids) suffer from segregation of parental alleles that breaks up co‐adapted gene complexes. Consequently, for the F1 to be fitter than later‐generation hybrids, the cost of dominance experienced to a greater extent by the F1, must be less than the cost of segregation experienced by these later‐generation hybrids. A simple generalization of Equation 18 (eq. 53) shows that hybrid breakdown between well adapted parental lines will occur only if:

(21) $$V_{\delta }<\frac{4h\left(1-h\right)-p_{12}}{1-p_{12}}.$$

where *h* is the hybrid index (the proportion of divergent alleles that come from one the parental lines) and *p*_12_ is the interpopulation heterozygosity. It follows that F2 breakdown will occur whenever $V_{\delta }<1$, since 4h(1−h)≈1 for an F2. And the same weak bound follows from observations of selection for increased heterozygosity in recombinant hybrids (Lindtke et al., 2012; Simon et al., 2018; Thompson et al., 2022). Breakdown among backcross hybrids sets a stronger bound because it occurs only if $V_{\delta }<p_{12}$ (since $4h\left(1-h\right)=p_{12}\left(2-p_{12}\right)$ for any backcross), and $p_{12}=1/2$ on average for the first backcross.

While the upper bounds on $V_{\delta }$ are not tight, existing data are at least consistent with a world where dominance effects are non‐zero but small (i.e., where $0<V_{\delta }\ll 1$). In this case, simple additive phenotypic models would yield good predictions for later‐generation recombinant hybrids (Fraïsse et al., 2016; Simon et al., 2018), even when dominance qualitatively affects outcomes for the F1.

## AUTHOR CONTRIBUTIONS

A.D.M. and K.A.T. conceived the original idea of the study, performed simulations, and interpreted results. J.J.W. and H.S. expanded these with analytical predictions and individual‐based simulations. All authors discussed the results, gave critical feedback, and contributed to the final manuscript.

## CONFLICT OF INTEREST STATEMENT

The authors declare no conflict of interest.

## DATA ARCHIVING

All simulation data, code used to generate this data, and scripts used for processing and generating figures can be found at https://doi.org/10.5061/dryad.2bvq83bt9.

1

Associate Editor: L. Fromhage

Handling Editor: T. Chapman

## Supporting information

Figure S1: Size‐dependent distribution of dominance coefficients
Figure S2: Transgressive F1 trait variation can increase or decrease with genetic divergence
Figure S3: The F1 clock tick rate increases steadily with $V_{\delta_{\mathrm{mut}}}$ under various model parameters
Figure S4: Directional dominance leads to a transient fitness increase and then a permanent decrease
Figure S5: Major patterns of F1 fitness for simulated F1 hybrids using the dominance function illustrated in Fig. S1.
Figure S6: The probability of F1 heterosis in a novel environment depends on the number of traits, and the parental phenotypes
Figure S7: Lucky beneficial effects of dominance in novel environments, with variable levels of mutational dominance
Figure S8: Cartoon of fitness pattern that could be caused either by dominance or by different selection regimes between parental environments
Figure S9: Putative examples of fitness pattern indicative of phenotypic dominance
Figure S10: Derivation of the expected levels of log fitness asymmetry between cross directions with variable phenotypic dominance
