# Supplementary material for: The diverse effects of phenotypic dominance on hybrid fitness
Source: Evolution. 2022 Oct 27;76(12):2846–63. doi: 10.1111/evo.14645 (PMC10092378; doi:10.1111/evo.14645)
Supplement: Supplementary file 1 — Figure S1: Size‐dependent distribution of dominance coefficients Figure S2: Transgressive F1 trait variation can increase or decrease with genetic divergence Figure S3: The F1 clock tick rate increases steadily with Vδmut under various model parameters Figure S4: Directional dominance leads to a transient fitness increase and then a permanent decrease Figure S5: Major patterns of F1 fitness for simulated F1 hybrids using the dominance function illustrated in Fig. S1. Figure S6: The probability of F1 heterosis in a novel environment depends on the number of traits, and the parental phenotypes Figure S7: Lucky beneficial effects of dominance in novel environments, with variable levels of mutational dominance Figure S8: Cartoon of fitness pattern that could be caused either by dominance or by different selection regimes between parental environments Figure S9: Putative examples of fitness pattern indicative of phenotypic dominance Figure S10: Derivation of the expected levels of log fitness asymmetry between cross directions with variable phenotypic dominance [file EVO-76-2846-s001.pdf]

Supplementary Figures

Figure S1: Size-dependent distribution of dominance coefficients

Figure S2: Transgressive F1 trait variation can increase or decrease with genetic divergence

Figure S3: The F1 clock tick rate increases steadily with $V_{\delta_{\mathrm{mut}}}$ under various model parameters

Figure S4: Directional dominance leads to a transient fitness increase and then a permanent decrease

Figure S5: Major patterns of F1 fitness for simulated F1 hybrids using the dominance function illustrated in Fig. S1.

Figure S6: The probability of F1 heterosis in a novel environment depends on the number of traits, and the parental phenotypes

Figure S7: Lucky beneficial effects of dominance in novel environments, with variable levels of mutational dominance

Figure S8: Cartoon of fitness pattern that could be caused either by dominance or by different selection regimes between parental environments

Figure S9: Putative examples of fitness pattern indicative of phenotypic dominance

Figure S10: Derivation of the expected levels of log fitness asymmetry between cross directions with variable phenotypic dominance

Appendix 1: Derivations

Appendix 2: Simulation procedure

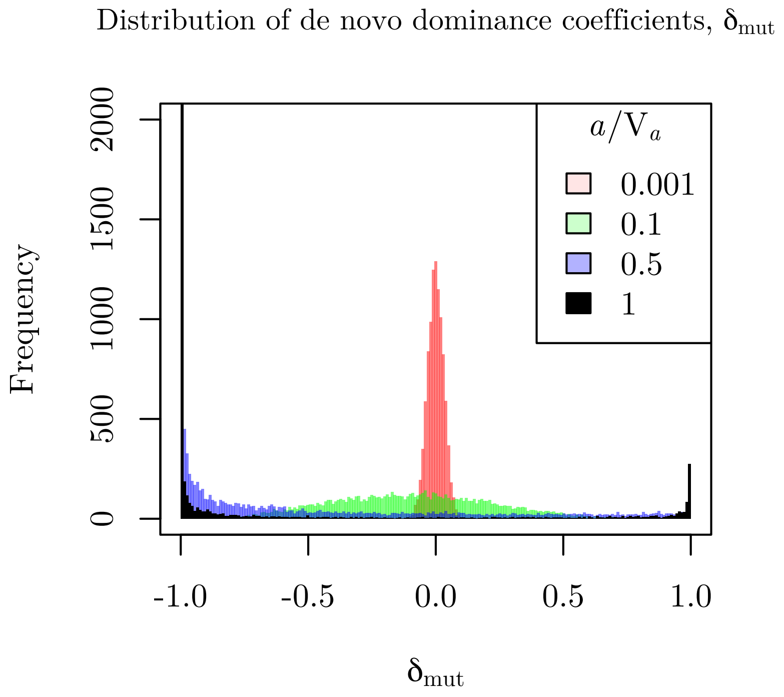

Figure S1: Size-dependent distribution of dominance coefficients. The distribution of dominance coefficients for new mutations as used in the simulations reported in Figures 2c, 5b and Figure S5. The heterozygous effect of a mutation on a given trait is $a_{\mathrm{mut}}(1 + \delta_{\mathrm{mut}})$. Here, a_{mut} is the mutation's additive effect, which is normally distributed with vanishing mean and standard deviation $V_{a_{\mathrm{mut}}}$ (eq. 60). The dominance coefficient, δ_{mut}, is drawn from a beta distribution, whose parameters vary with the size of of the additive effect according to eqs. 61-62. The aim is to reflect empirical observations that lethal or highly deleterious mutations are often recessive, while small-effect mutations are more likely to be semi-dominant (Orr, 1991; Manna et al., 2011).

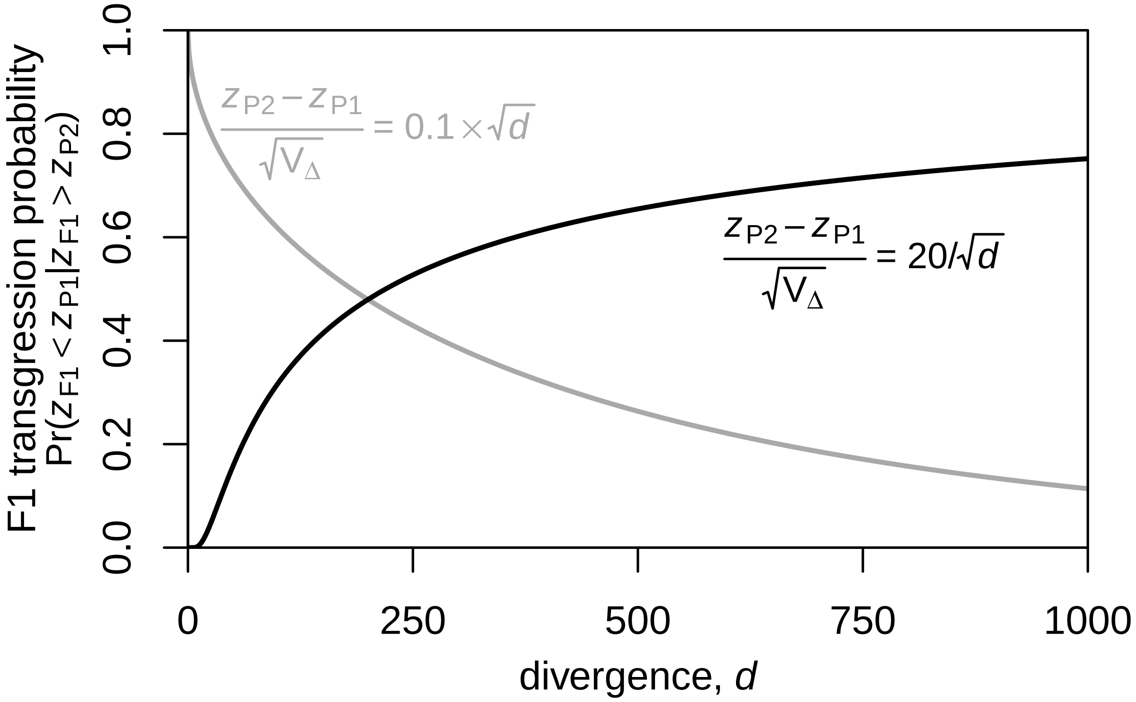

Figure S2: Transgressive F1 trait variation can increase or decrease with genetic divergence. Curves show the probability that the F1 trait value lies outside of the range of values in the two parental lines (eq. 48). The variance in the dominance coefficients, V_Δ increases linearly with genomic divergence, d (e.g. eqs. 6-7). If the divergence in the parental phenotypes also increases with d (e.g. due to directional selection) then the probability of transgressive variation decreases with d (grey curve). If the parental phenotypes remain roughly constant, e.g., due to effective stabilizing selection, then the probability of transgression increases (black curve). Both regimes have been observed in data (Stelkens and Seehausen, 2009).

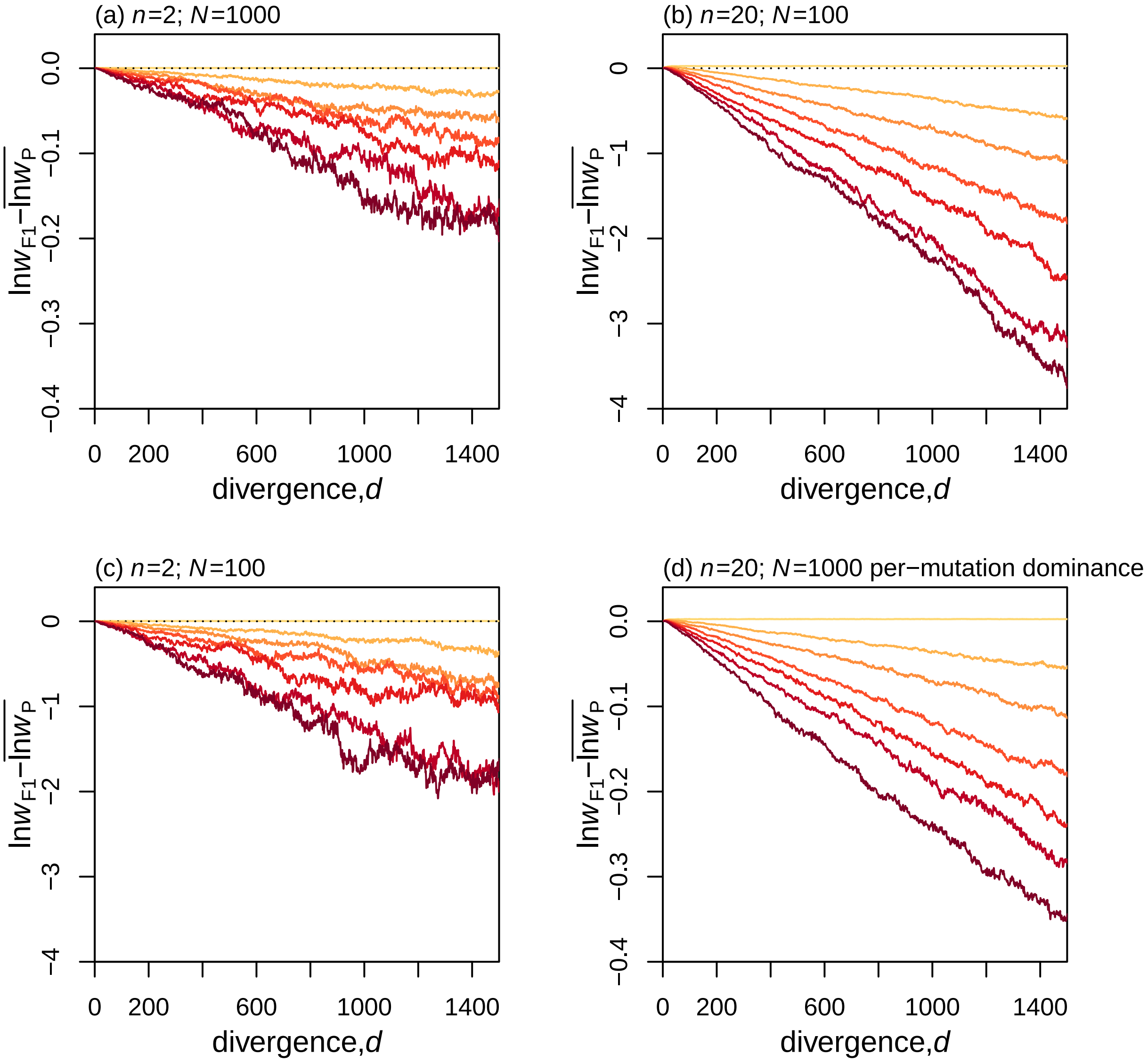

Figure S3: The F1 clock tick rate increases steadily with $V_{\delta_{\rm mut}}$ under various model parameters. **(a)-(c)**: Results replicate those shown in Figure 3b, except that the simulation parameters differed in terms of the number of traits (a), the population size (b), or both (c). In all these cases, the slopes are proportional to $V_{\delta_{\rm mut}}$. **(d)**: Results also hold in simulations where each mutation had a single value of $\delta_{\rm mut}$ applying to all n traits; such that heterozygous effects differed from the homozygous effects in their length but not in their direction. This shows that the effects of dominance reported here do not depend on the variation in direction.

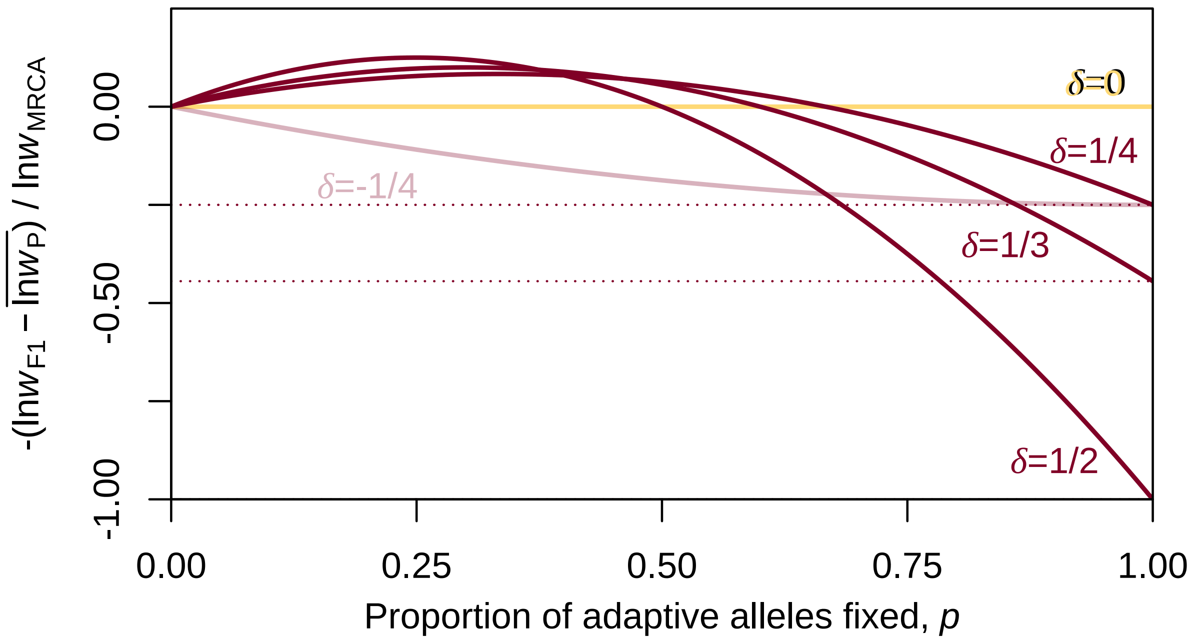

Figure S4: Directional dominance leads to a transient fitness increase and then a permanent decrease. Log F1 fitness relative to current and pre-adaptation parental fitness when parental lines are approaching a new optimum through the fixation of identical substitutions with equally sized additive effects and dominance effects (see eq. 38). The size of the dominance coefficients is denoted δ. The yellow line $\delta = 0$ represents additivity, the dark red curves represent dominance coefficients of different magnitudes, and the faint red curve represents adaptation via recessive substitutions for comparison. With dominance, F1 fitness first exceeds parental fitness due to the directional dominance overshoot, but as parental lines get closer to the new optimum this overshoot becomes deleterious and F1 fitness declines. The horizontal dotted lines give the predicted overshoot when the optimum has been reached.

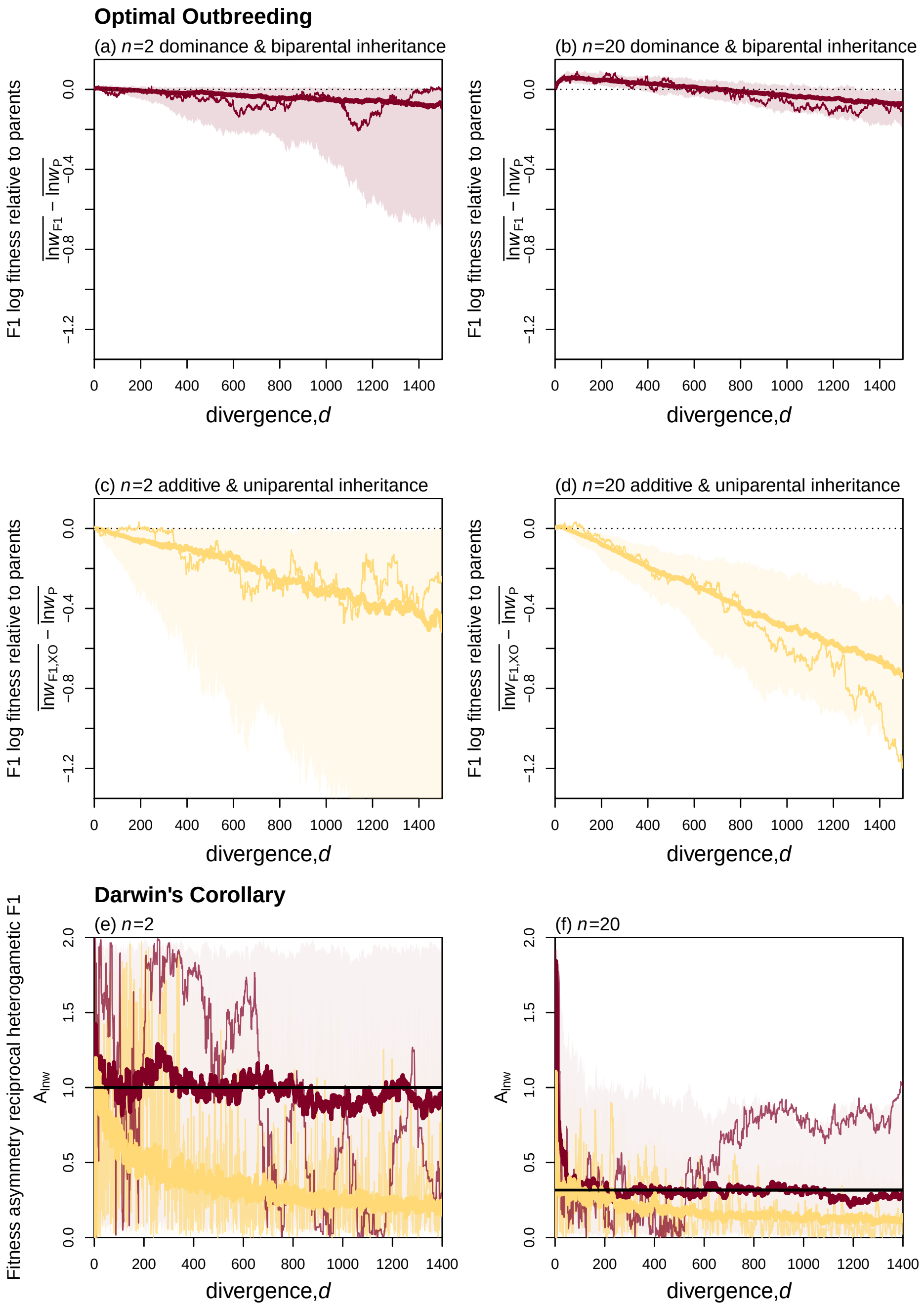

Figure S5: Major patterns of F1 fitness for simulated F1 hybrids using the dominance function illustrated in Fig. S1. Simulation results are reported comparing phenotypic additivity (yellow curves) to phenotypic dominance, using the dominance function shown in Fig. S1, where mutations of larger effect had more extreme levels of dominance (dark red curves). Results correspond to **(a)-(b)**: Fig. 2c; **(c)-(d)**: Fig. 5b; **(d)-(e)**: Fig. 6c-d. In all cases, the average over 100 replicates (thicker solid lines) is compared to a single randomly chosen replicate (thinner lines), to illustrate the stochasticity. Heterogametic F1 in (c)–(f) used ($x = \frac{1}{3}$).

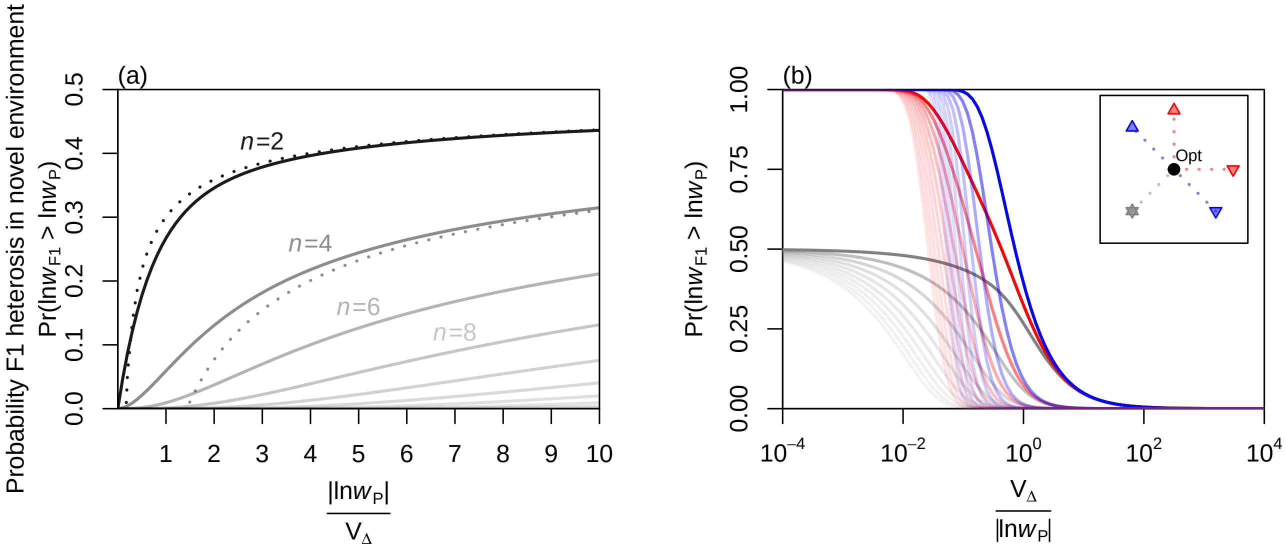

Figure S6: The probability of F1 heterosis in a novel environment depends on the number of traits, and the parental phenotypes. Panel **(a)** and gray lines in **(b)** represent the scenario from Figure 4a, when the two parental lines have similar and equally maladapted phenotypes. **(a)**: a replication of Figure 4b, but without log transformation on the x-axis, highlighting the decline in the probability of heterosis with n. **(b)**: the probability of heterosis increases if the parents are maladapted on different traits (red curves), or in opposite phenotypic directions (blue curves). The positions of the parents relative to the optimum are shown in the inset. Colour intensity indicates the value of n which ranges from 2 (bright lines) to 20 (faint lines), and in all cases, the probability of heterosis declines steeply with n and with $V_\Delta/|\ln w_\mathrm{P}|$. The functions plotted are described in Appendix 1.

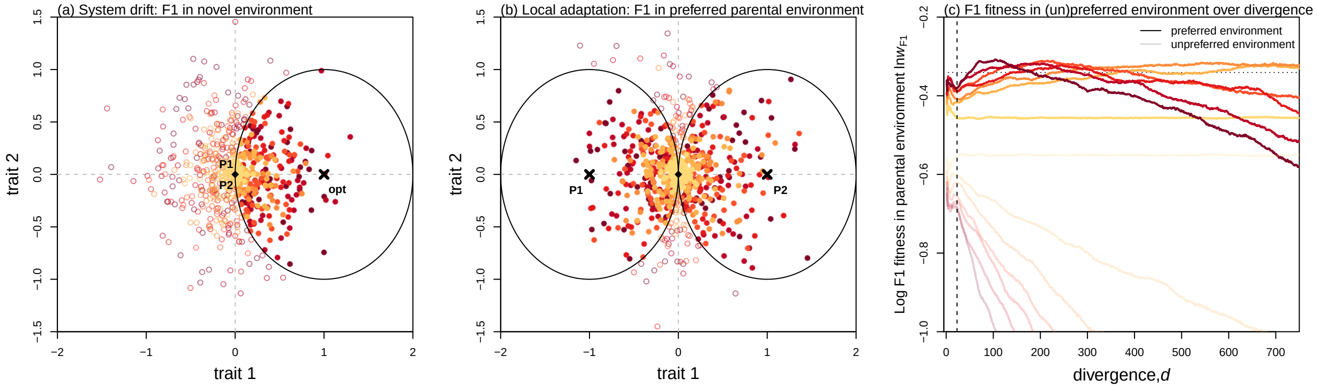

Figure S7: Lucky beneficial effects of dominance in novel environments, with variable levels of mutational dominance. **(a)–(b)**: Simulated F1 hybrids after 50 divergent substitutions with different levels of variation in dominance show "Lucky" beneficial effects in a new environment. **(c)**: Simulations of the expected log fitness of the F1 between locally adapted parents, scored in the preferred parental habitat, to which the F1 is better adapted (solid lines) or the unpreferred parental habitat, to which it is less adapted (faint lines). These results match Figure 4d, but include results for all seven different levels of variance among the dominance coefficients of the new mutations, $V_{\delta_{\mathrm{mut}}}$. Colours for the $V_{\delta_{\mathrm{mut}}}$ values match those in Figure 3, with darker colours indicating higher mutational variance.

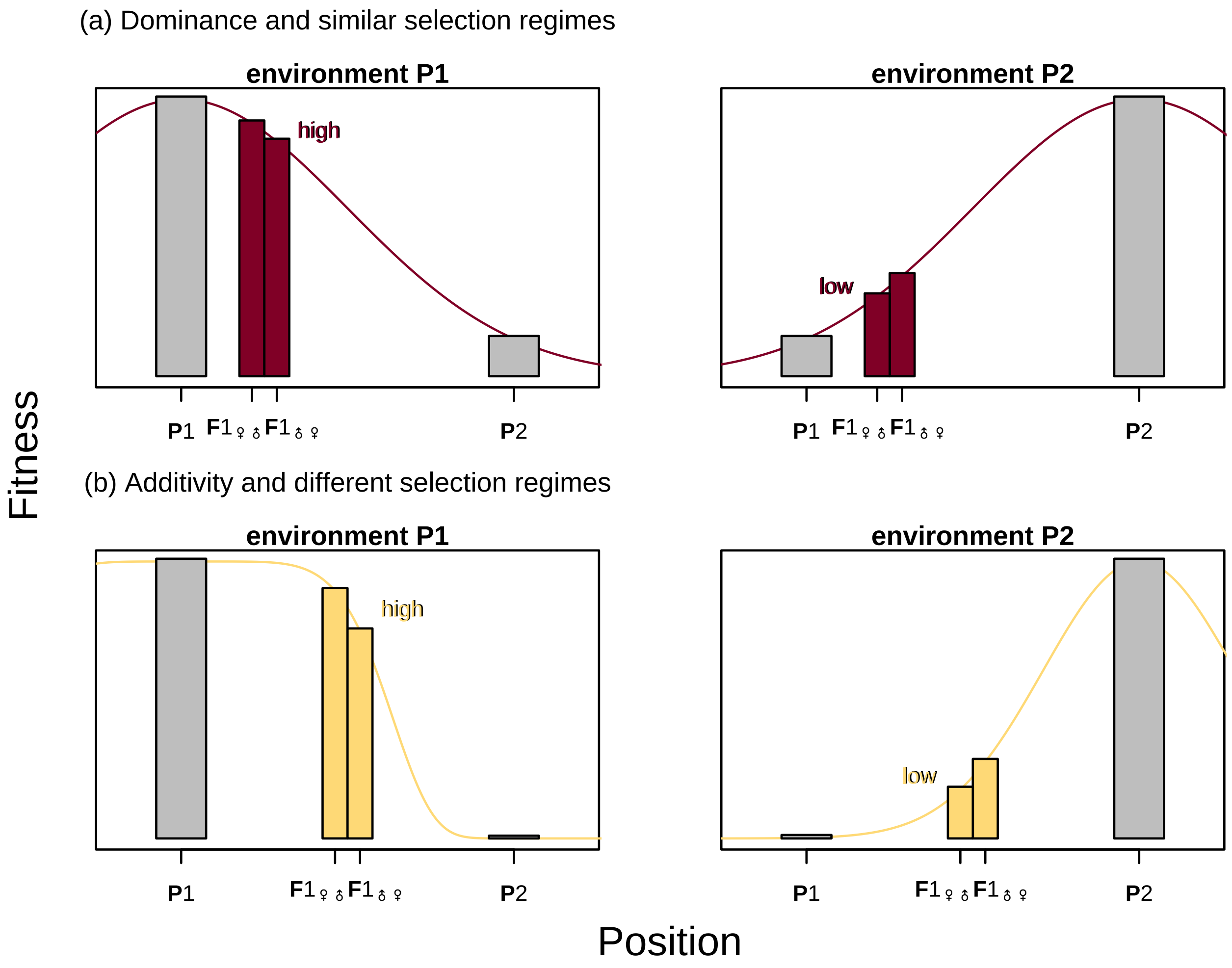

Figure S8: Cartoon of fitness pattern that could be caused either by dominance or by different selection regimes between parental environments In this example, both reciprocal F1 between locally adapted parents have high fitness in one and low fitness in the other parental environment. When the strength of selection and curvature of the fitness landscape are the same in both environments (top row), this fitness pattern is indicative of phenotypic dominance. However, different selection regimes in the two parental environments can also explain this pattern under additivity (bottom row), and therefore it is not a straightforward test of dominance.

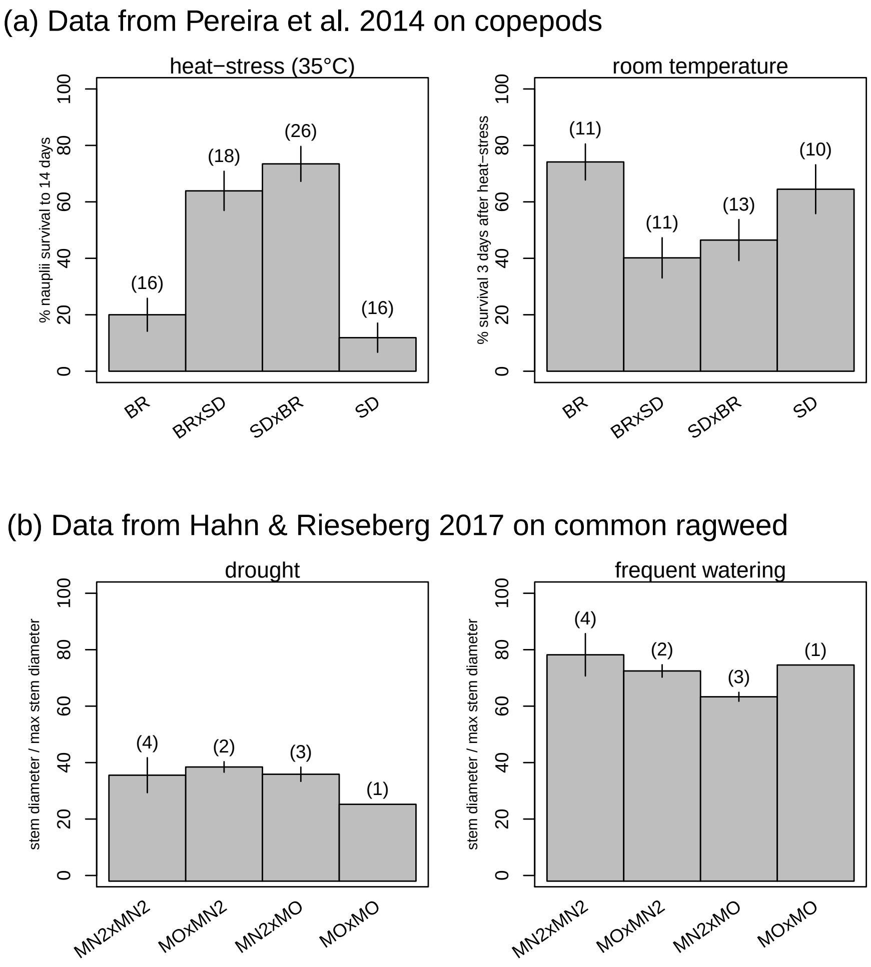

Figure S9: Putative examples of fitness pattern indicative of phenotypic dominance. (a): Data from Bird rock ('BR') and San Diego ('SD') populations of copepod *Tigriopus californicus* and their reciprocal F1 hybrids ('BRxSD' and 'SDxBR') from Pereira et al. (2014). Left and right plot show percentage of nauplii survival after 14 days under control conditions, and percentage adult survival 3 days after heat-stress treatment at 35°C respectively. The height of the bars indicates the mean across replicates, the vertical lines indicate one standard error, and the number in brackets is the number of clutches (left) or the number of replicates containing 10 individuals each (right). **(b):** Data from common ragweed (*Ambrosia artemisiifolia*) intrapopulation crosses between individuals from Missouri ('MOxMO') and Ontario ('MN2xMN2'), and their reciprocal interpopulation hybrids ('MOxMN2' and 'MN2xMO') from Hahn and Rieseberg (2017). Plotted is the stem diameter divided by the maximum stem diameter in the whole dataset under drought and control conditions for the left and right plot respectively. The height of the bars indicates the mean across individuals in that category, the vertical lines indicate one standard deviation, and the sample size for that category is printed in brackets.

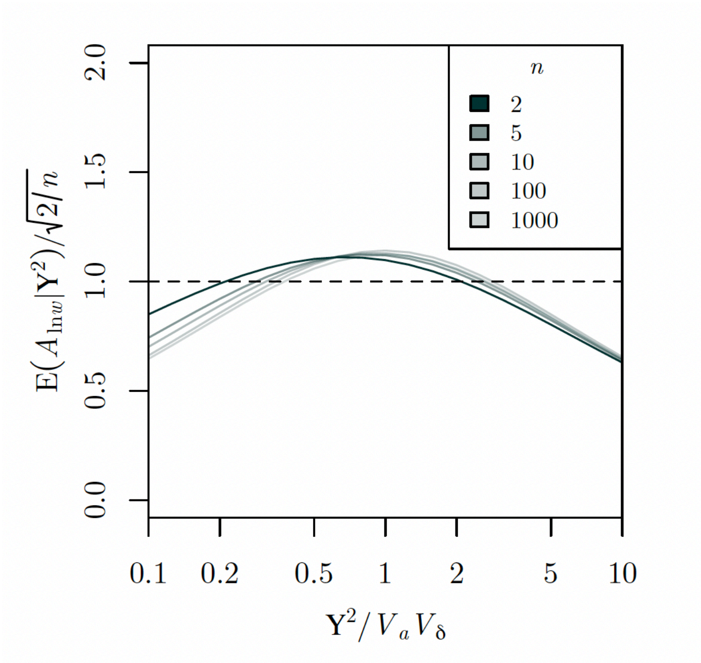

Figure S10: Derivation of the expected levels of log fitness asymmetry between cross directions with variable phenotypic dominance. The expected value of eq. 56 is shown for various values of n, namely (dark-to-light) $n = 2, 5, 10, 100$ and 1000. The expectation (evaluated numerically) is close to the value of the crude approximation $\sqrt{2/n}$ (eq. 20), unless the quantity $\Upsilon^2/V_a V_\delta$ deviates greatly from unity. This quantity compares the deviations due to dominance, and the deviations due to lack of autosomal co-adaptation with the absent paternal X chromosome (see Figure 5a), and depends mainly on the variance in the dominance effects and the proportion of uniparentally inherited loci (eq. 58).

Appendix 1: Derivations

In this Appendix, we derive the analytical results in the main text. The relevant main text equations are listed next to each section. We present results under a quadratic fitness function, but in case of higher fitness landscape curvature, our results apply to the squared distance to the optimum instead. For some datasets, the curvature can be estimated (eg. if F1 fitness has been measured across a range of parental divergence, assuming selection was predominantly stabilizing) and this can be used to transform fitness measures to obtain squared distances.

1 F1 fitness with biparental inheritance

Many results in the main text are given in terms of the total dominance deviations on each trait, that is, the Δ_i as defined in eq. 4. The strongest assumptions that we use are the normality and isotropy of the Δ_i (i.e. that all Δ_i have equal variances). However, neither assumption is required for all results, and the assumption of isotropy is made largely for simplicity.

1.1 Expected log fitness (eqs. 3–11)

To obtain the expected log fitness of the midparent given in eq. 3, we use the definition of cosine similarity that captures the similarity between two vectors, which in our case are the displacements of the two parental lines from the optimum.

$$\cos\theta \equiv \frac{\sum_{i=1}^{n}\left(z_{\mathrm{P1},i}-o_i\right)\left(z_{\mathrm{P2},i}-o_i\right)}{\sqrt{\sum_{i=1}^{n}\left(z_{\mathrm{P1},i}-o_i\right)^2}\sqrt{\sum_{i=1}^{n}\left(z_{\mathrm{P2},i}-o_i\right)^2}}$$

$$= \frac{\sum_{i=1}^{n}\left(z_{\mathrm{P1},i}-o_i\right)\left(z_{\mathrm{P2},i}-o_i\right)}{\sqrt{\ln w_{\mathrm{P1}} \times \ln w_{\mathrm{P2}}}} \tag{22}$$

With the geometric mean of two numbers defined as the square root of their product, we can replace $\sqrt{\ln w_{\mathrm{P1}} \times \ln w_{\mathrm{P2}}}$ with $-\overline{\ln w_{\mathrm{P}}}^{(g)}$ to obtain eq 3. Note that because the substitution effects are defined with respect to the P1 genotype, the log fitness of the midparent can also be expressed

in terms of the substitution effects as $\ln w_{\mathrm{MP}} = \sum_{i=1}^{n}\left(z_{\mathrm{P1},i} + \sum_{j=1}^{d} a_{ij} - o_i\right)^2$.

To calculate the expected log fitness of the F1 we need to consider the heterozygous effects of the substitutions with dominance

$$\begin{aligned}\ln w_{\mathrm{F1}} &\equiv -\sum_{i=1}^{n}\left(z_{\mathrm{P1},i} + \sum_{j=1}^{d} a_{ij}\left(1+\delta_{ij}\right) - o_i\right)^2 \\ &= \ln w_{\mathrm{MP}} - \sum_{i=1}^{n}\left(\Delta_i^2 + 2\left(z_{\mathrm{MP},i} - o_i\right)\Delta_i\right)\end{aligned} \tag{23}$$

where we have used the definition of Δ_i provided in eq. 4. After rotating the axes such that the midparent falls short of the optimum only on trait 1 such that $z_{\mathrm{MP},i} = 0$ for all $i \neq 1$, the cross product simplifies to $-2\sqrt{|\ln w_{\mathrm{MP}}|}\Delta_1$. Then we can separate trait 1 from the others to obtain eq. 5.

So far, results are exact. However, we will usually not know the Δ_i and therefore we would like to know what its expected value and variance are under different scenarios of parental divergence. In other words, we want to find the expected log fitness of the F1, where the expectation is across realisations of the divergence history of the parental lines, i.e. over the complete sets of a_{ij} and δ_{ij} that give rise to different Δ_i. We begin by expanding the brackets in eq. 5:

$$\ln w_{\mathrm{F1}} = \ln w_{\mathrm{MP}} - 2\sqrt{|\ln w_{\mathrm{MP}}|}\Delta_1 - \sum_{i=1}^{n}\Delta_i^2 \tag{24}$$

Then we take expectations over the possible values of the dominance deviations, to account for their relative independence of the form of selection acting on the parental lines.

$$E\left(\ln w_{\mathrm{F1}}|\ln w_{\mathrm{MP}}\right) = \ln w_{\mathrm{MP}} - 2\sqrt{|\ln w_{\mathrm{MP}}|}E\left(\Delta_1\right) - \sum_{i=1}^{n} E\left(\Delta_i^2\right) \tag{25}$$

Equations 9–10 follow directly, if we focus only on trait 1.

1.1.1 Stabilizing selection (eqs. 6–8)

Next, if there is no directional dominance, i.e., if all $E(\Delta_i) = 0$, then:

$$E\left(\ln w_{\mathrm{F1}} | \ln w_{\mathrm{MP}}\right) = \ln w_{\mathrm{MP}} - \sum_{i=1}^{n} \mathrm{Var}\left(\Delta_i\right), \qquad E(\Delta_i) = 0 \tag{26}$$

and so eq. 6 follows directly if we use the notation:

$$V_\Delta \equiv \frac{1}{n} \sum_{i=1}^{n} \mathrm{Var}\left(\Delta_i\right) \tag{27}$$

For eq. 7, we use equivalent notation to refer to the effects of the individual substitutions, which under stabilizing selection, we can treat as increments in a random walk, with variance

$$V_{a\delta} \equiv \frac{1}{n} \sum_{i=1}^{n} \mathrm{Var}\left(a_i \delta_i\right) \tag{28}$$

where $a_i\delta_i$ is a randomly chosen dominance effect on trait i, and the expectations are over realizations of the evolutionary process. We can also define an equivalent quantity for the additive effects.

$$V_a \equiv \frac{1}{n} \sum_{i=1}^{n} \mathrm{Var}\left(a_i\right) \tag{29}$$

where a_i is a randomly chosen additive effect on trait i.

V_δ cannot generally be defined in the same way, except in the special case of isotropy, and no covariance between the squares of the additive and dominance effects. In this case $V_\delta = \mathrm{Var}\left(\delta_i\right)$ such that it has a more intuitive definition, equal to the variability across realisations of divergence in the dominance coefficients of fixed substitutions. We can see this if we write $V_{a\delta}$ in terms of moments of additive effects and dominance coefficients, where similar to the above we use δ_i to refer to the dominance coefficient on trait i of a randomly chosen substitution.

$$V_{a\delta} \equiv \frac{1}{n}\sum_{i=1}^{n} E\left(a_i^2\delta_i^2\right) - E^2\left(a_i\delta_i\right) \tag{30}$$

With exchangeable parental lines, and recalling that the effects of a substitution are defined with respect to P1, irrespective of whether they are ancestral or derived, it follows that $E\left(a_i\right) = E\left(\delta_i\right) = 0$, such that

$$E\left(a_i\delta_i\right) = \mathrm{Cov}\left(a_i, \delta_i\right) \tag{31}$$

$$E\left(a_i^2\delta_i^2\right) = \mathrm{Cov}\left(a_i^2, \delta_i^2\right) + \mathrm{Var}\left(a_i\right)\mathrm{Var}\left(\delta_i\right) \tag{32}$$

Without directional dominance $\mathrm{Cov}\left(a_i, \delta_i\right) = 0$, and whenever larger substitutions have no tendency to have more extreme dominance coefficients, then $\mathrm{Cov}\left(a_i^2, \delta_i^2\right) = 0$, and we get

$$V_{a\delta} = \frac{1}{n}\sum_{i=1}^{n} \mathrm{Var}\left(a_i\right)\mathrm{Var}\left(\delta_i\right), \qquad \mathrm{Cov}\left(a_i, \delta_i\right) = \mathrm{Cov}\left(a_i^2, \delta_i^2\right) = 0 \tag{33}$$

Under isotropy, the distribution of substitutions is the same across traits, so in this case we obtain the more intuitive interpretation $V_\delta = \mathrm{Var}\left(\delta_i\right)$ and also $V_a = \mathrm{Var}\left(a_i\right)$, where both are a constant. Note, however, that data do not support the assumption that $\mathrm{Cov}\left(a_i^2, \delta_i^2\right) = 0$ (Orr, 1991; Manna et al., 2011).

1.1.2 Directional selection (eqs. 9–11)

Under directional selection on trait 1, we expect additive effects in the direction of the optimum to be more dominant. Therefore, for these adaptive substitutions (denoted by an asterisk $*$), $\mathrm{Cov}\left(a_1^*, \delta_1^*\right)$ will not vanish. However, we can obtain results in a simplified case in which the initial bout of adaptation took place in both parental lineages, taking them both from an old optimum to a new optimum, and where these optima differed only on trait 1. We will assume that the adaptation took place in exactly d_{dir} substitutions, and that these substitutions affected only trait

1. We will further assume that their additive effects and dominance coefficients covary in sign, but not in size, such that $\mathrm{Cov}\left(|a_1^*|, |\delta_1^*|\right) = 0$. In other words, large adaptive substitutions are no more likely to be dominant or recessive than small adaptive substitutions, but substitutions pointing from the MRCA towards the optimum (adaptive substitutions that are derived in P2) have $\delta_1^*, a_1^* > 0$ whereas substitutions pointing towards the MRCA and away from the optimum (adaptive substitutions derived in P1) have $\delta_1^*, a_1^* < 0$.

Taken together, the chain of adaptive substitutions runs from the new optimum to the old optimum and then back, and so its total length is $2d_{\mathrm{dir}}\,|a_1^*|$, and we have $d_{\mathrm{dir}}\,|a_1^*| = \sqrt{|\ln w_{\mathrm{MRCA}}|}$, where w_{MRCA} is the fitness of ancestral genotype (adapted to the old optimum), in the environment characterized by the new optimum. With these assumptions, we can now derive the moments of Δ_1 after both the adaptation, and a further period of $d - d_{\mathrm{dir}}$ substitutions, accrued under stabilizing selection:

$$\begin{aligned} E\left(\Delta_1\right) &= E\left[\sum_{j=1}^{d_{\mathrm{dir}}} a_{1j}^*\delta_{1j}^* + \sum_{j=d_{\mathrm{dir}}+1}^{d} a_{1j}\delta_{1j}\right] \qquad (34) \\ &= d_{\mathrm{dir}} E\left[a_1^*\delta_1^*\right] \\ &= \sqrt{|\ln w_{\mathrm{MRCA}}|}\,E\left[|\delta_1^*|\right] \end{aligned}$$

$$\begin{aligned} E\left(\Delta_1^2\right) &= E\left[\left(\sum_{j=1}^{d_{\mathrm{dir}}} a_{1j}^*\delta_{1j}^* + \sum_{j=d_{\mathrm{dir}}+1}^{d} a_{1j}\delta_{1j}\right)^2\right] \\ &\approx d_{\mathrm{dir}}^2 E\left[\left(a_1^*\delta_1^*\right)^2\right] + \left(d - d_{\mathrm{dir}}\right) E\left[\left(a_1\delta_1\right)^2\right] \\ &= |\ln w_{\mathrm{MRCA}}|\, V_{\delta_{\mathrm{dir}}} + \left(d - d_{\mathrm{dir}}\right) \mathrm{Var}\left(a_1\delta_1\right) \qquad (35) \end{aligned}$$

where

$$V_{\delta_{\mathrm{dir}}} \equiv E\left[\delta_1^{*2}\right] \qquad (36)$$

Here, we used the random-walk assumptions, described above, for the substitutions accrued

after the adaptation. Equation 35 also uses the stronger assumption that covariance between the dominance effects of the adaptive substitutions can be ignored. After making these assumptions, eq. 11 in the main text follows directly.

We can also give a more complete result that applies during the ongoing adaptation to the new optimum. During the adaptation phase i.e. before the parental lines have reached the optimum, we use $p = \frac{z_{\mathrm{MRCA}} - z_{\mathrm{MP}}}{z_{\mathrm{MRCA}}}$ to denote the proportion of the distance to the new optimum the parental lines have already travelled. Considering only the trait under directional selection, we find

$$E\left(\ln w_{\mathrm{F1}}\right) = \ln w_{\mathrm{MP}} - 2p(1-p)E\left[\left|\delta_1^*\right|\right]\left|\ln w_{\mathrm{MRCA}}\right| - p^2\left|\ln w_{\mathrm{MRCA}}\right| V_{\delta_{\mathrm{dir}}} \tag{37}$$

For the overshoot of the F1, we find

$$E\left(\frac{\ln w_{\mathrm{F1}} - \ln w_{\mathrm{MP}}}{\ln w_{\mathrm{MRCA}}}\right) = -p^2 V_{\delta_{\mathrm{dir}}} - 2p(1-p)E\left[\left|\delta_1^*\right|\right] \tag{38}$$

These expressions are plotted in Figure S4 for substitutions with dominance coefficients of identical size $|\delta|$.

1.2 Variance in F1 log fitness (eq. 12)

To calculate eq. 12 we also require the variance in log F1 fitness. Its behaviour is easiest to see if we assume normality and independence of the Δ_i. In this case, we find:

$$\mathrm{Var}\left(\ln w_{\mathrm{F1}}\right) = E\left(\mathrm{Var}\left(\ln w_{\mathrm{F1}} \left| \ln w_{\mathrm{MP}}\right.\right)\right) + \mathrm{Var}\left(E\left(\ln w_{\mathrm{F1}} \left| \ln w_{\mathrm{MP}}\right.\right)\right)$$

$$= 4E\left(\ln w_{\mathrm{MP}}\right) V_{\Delta_1} + 2\sum_{i=1}^{n} V_{\Delta_i}^2 + \mathrm{Var}\left(\ln w_{\mathrm{MP}}\right), \qquad E(\Delta_i) = 0 \tag{39}$$

If the midparent remains well adapted, but the dominance deviations are large, then the second term will come to dominate. Because V_Δ grows with the divergence, this will typically hold at large divergences, when $d \gg 1$, and so

$$\mathrm{Var}\,(\ln w_{\mathrm{F1}}) \approx 2nV_{\Delta}^2, \qquad E(\Delta_i) = 0, d \gg 1 \tag{40}$$

Using the same limit in eq. 6 yields eq. 12.

1.3 F1 fitness in preferred parental environment (eqs. 14, 15)

When the parental lines are adapted to two different optima, then the F1 will tend to be fitter in one of the two parental environments (i.e. its "preferred" environment). In that environment, we can write its log fitness using a small modification of eq. 24:

$$\ln w_{\mathrm{F1}_{\mathrm{pref}}} = -\left(\sqrt{|\ln w_{\mathrm{MP}}|} - |\Delta_1|\right)^2 - \sum_{i=2}^{n} \Delta_i^2$$

$$= \ln w_{\mathrm{MP}} - \sum_{i=1}^{n} \Delta_i^2 + 2\sqrt{|\ln w_{\mathrm{MP}}|}|\Delta_1| \tag{41}$$

If we assume that the parental lines are each optimal in one of the habitats, then distance of midparent from either optimum is half of the distance between the optima, such that $\ln w_{\mathrm{MP}} = \ln w_{\mathrm{P}}/4$, where $\ln w_{\mathrm{P}}$ is the log fitness of the maladapted parental line in any given habitat. Next, we assume that Δ_1 is normal, with a vanishing mean, so that $E(|\Delta_1|) = \sqrt{\mathrm{Var}(\Delta_1)2/\pi}$. Equation 14 then follows directly if we assume equal variances for the dominance deviations on all traits, so that $\mathrm{Var}(\Delta_1) = V_{\Delta}$.

To derive equation 15, we differentiate eq. 14 by V_{Δ}, and set to zero. This yields the value of V_{Δ} that maximises the expected log fitness. Substituting that value into eq. 14 yields eq. 15.

As with eq. 39, we can also derive the variance in $\ln w_{\mathrm{F1}_{\mathrm{pref}}}$ using the assumptions of normality, independence and vanishing means for the Δ_i:

$$\mathrm{Var}\left(\ln w_{\mathrm{F1}_{\mathrm{pref}}}\right) = 2nV_\Delta^2 - 4E\left(\ln w_{\mathrm{MP}}\right)V_{\Delta_1}\left(1-2/\pi\right) - 4E\left(\sqrt{|\ln w_{\mathrm{MP}}|}\right)\sqrt{V_{\Delta_1}2/\pi}$$

$$+ \mathrm{Var}\left(\ln w_{\mathrm{MP}} + 2\sqrt{|\ln w_{\mathrm{MP}}|}\sqrt{V_{\Delta_1}2/\pi}\right) \quad (42)$$

At large divergences, the first term will come to dominate, supporting the assertion in the main text that eq. 12 still holds for $\ln w_{\mathrm{F1}_{\mathrm{pref}}}$.

1.4 Probability of lucky heterosis (eq. 13)

Now let us consider the F1 between phenotypically identical parental lines, that are both maladapted. To calculate the probability of their F1 being heterotic (Fig 4b), we first note that, with isotropy, such that all $V_{\Delta_i} = V_\Delta$, then, from eq. 5, the quantity $|\ln w_{\mathrm{F1}}|/V_\Delta$ is the sum of n squared normal variables, one with a non-zero mean of $\sqrt{|\ln w_{\mathrm{MP}}|/V_\Delta}$. As such, this quantity will follow a non-central chi-squared distribution, with n degrees of freedom, and non-centrality parameter given by $|\ln w_{\mathrm{MP}}|/V_\Delta = |\ln w_{\mathrm{P}}|/V_\Delta$. Here, we have used the fact that the midparent will be identical to the two (identical) parental phenotypes. We can write the cumulative distribution function of this variable as

$$\mathrm{cdf}(|\ln w_{\mathrm{F1}}|/V_\Delta) = 1 - Q_{n/2}(\sqrt{|\ln w_{\mathrm{P}}|/V_\Delta}, \sqrt{|\ln w_{\mathrm{F1}}|/V_\Delta}) \quad (43)$$

where we have used the Marcum Q function (Marcum, 1950). The F1 will be heterotic if it is closer to the optimum than the midparent and so

$$\Pr\left(\ln w_{\mathrm{F1}} > \ln w_{\mathrm{P}}\right) = \Pr\left(\frac{\ln w_{\mathrm{F1}}}{V_\Delta} > \frac{\ln w_{\mathrm{P}}}{V_\Delta}\right)$$

$$= 1 - Q_{n/2}(\sqrt{|\ln w_{\mathrm{P}}|/V_\Delta}, \sqrt{|\ln w_{\mathrm{P}}|/V_\Delta}) \quad (44)$$

This is the exact result plotted in Figure 4b. This result can also be written in terms of modified Bessel functions of the first kind, as follows:

$$1 - Q_{n/2}(\sqrt{u}, \sqrt{u}) = \frac{1}{2} - e^{-u}\left[\frac{I_0(u)}{2} + \sum_{j=1}^{n/2-1} I_j(u)\right] \tag{45}$$

(Helstrom, 1995). Approximating these functions as $I_j(u) = \frac{e^u}{\sqrt{2\pi u}}$ (see, e.g. Guo et al., 2001), yields eq. 13 of the main text.

Figure S6 shows similar results when the parental lines are equally maladapted, but no longer phenotypically identical. To derive these results, we simply change the non-centrality parameter (i.e., the first argument of the Q function in eq. 44), which represents the position of the midparent. When the parents are maladapted in opposite directions, then the midparent matches the optimum, such that this argument is 0 (blue points and lines in Fig. S6). When the parents are maladapted in orthogonal directions (e.g., on different phenotypic traits), then this argument is $\sqrt{4|\ln w_{\mathrm{P}}|/V_{\Delta}}$ (red points and lines in Fig. S6).

1.5 Transgressive segregation in the F1

The procedure used in the previous section can also be used to calculate the probability of transgressive variation in a single trait: i.e., an F1 trait value that lies outside of the range of values among the parental lines (Stelkens and Seehausen, 2009). If there is no directional dominance, then the mean F1 trait value will be the midparental value, and its variance will be equal to the variance of the dominance deviations.

$$E(z_{\mathrm{F1}}) = \frac{z_{\mathrm{P2}} - z_{\mathrm{P1}}}{2} \tag{46}$$

$$\mathrm{Var}(z_{\mathrm{F1}}) = V_{\Delta}. \tag{47}$$

where we have ordered the parents by their trait value. Now, assuming normality, the proba-

bility of the F1 trait value lying outside of the parental range is

$$\Pr\left(z_{\mathrm{F1}} < z_{\mathrm{P1}} | z_{\mathrm{F1}} > z_{\mathrm{P2}}\right) = 1 - \mathrm{erf}\left(\frac{z_{\mathrm{P2}} - z_{\mathrm{P1}}}{2\sqrt{V_\Delta}}\right) \tag{48}$$

$$\approx \exp\left(-\frac{z_{\mathrm{P2}} - z_{\mathrm{P1}}}{2\sqrt{V_\Delta}}\right) \tag{49}$$

Because V_Δ grows linearly with d, the probability of transgressive variation will increase with d if the divergence between the parental phenotypes also increases with d (at least as fast as $\sqrt{d}$). If, by contrast, the difference between the parental traits remains static, then the probability of transgressive variation will decrease with d. This is illustrated in Figure S2. Both regimes were observed by Stelkens and Seehausen (2009).

2 Uniparental inheritance and recombinant hybrids

In this section, we present results for a more general type of hybrid, in which some loci are either hemizygous or homozygous. To do this, we will assume that the the parental lines are under stabilizing selection, as described above, such that the dominance deviations undergo a random walk in phenotypic space. We also use existing results for recombinant hybrids, conditional on the F1 fitness (Simon et al., 2018; Schneemann et al., 2020; De Sanctis et al., 2022).

2.1 Expected log fitness and bounds on V_δ (eqs. 16–18 and 21)

An arbitrary hybrid can be characterized by its hybrid index, $0 \leq h \leq 1$ (i.e. the proportion of divergent alleles from one of the parental lines) and by its heterozygosity, $0 \leq p_{12} \leq 1$ (the proportion of divergent loci carrying one allele from each parental line). Let us first begin by defining A_i as the analogue to Δ_i for the additive effects.

$$A_i = \sum_{j=1}^{d} a_{ij} \tag{50}$$

With this notation, the midparental log fitness can be written as

$$\ln w_{\rm MP} = \overline{\ln w_{\rm P}} + \sum_{i=1}^{n} A_i^2 \tag{51}$$

It then follows from eq. 3, that when the populations are maladapted from their optimum in random directions, such that $E(\ln w_{\rm MP}) = \frac{\overline{\ln w_{\rm P}}}{2}$, then

$$E\left(\sum_{i=1}^{n} A_i^2\right) = -\frac{\overline{\ln w_{\rm P}}}{2} \tag{52}$$

Now, using the assumptions discussed above for stabilizing selection, we can combine eq. 52 with the published results, to yield the expected log fitness of an arbitrary hybrid.

$$\begin{aligned} E(\ln w_{\rm H}) &= \overline{\ln w_{\rm P}}\,(1-2h(1-h)) - p_{12}E\left(\sum_{i=1}^{n}\Delta_i^2\right) - (4h(1-h)-p_{12})\,E\left(\sum_{i=1}^{n}\sum_{j=1}^{d} a_{ij}^2\right) \\ &= \overline{\ln w_{\rm P}}\,(1-2h(1-h)) - p_{12}dnV_\delta V_a - (4h(1-h)-p_{12})\,dnV_a \end{aligned} \tag{53}$$

Note that eq. 53 contains three terms, the second capturing the deleterious effects of dominance, and the third capturing the deleterious effects of lack of coadaptation among the additive effects. Equation 53 also reduces to results in the main text for a fully heterozygous F1, for which $h = 1/2$ and $p_{12} = 1$. Equation 21 follows from comparing this F1 to the arbitrary hybrid, when parents are well adapted ($\overline{\ln w_{\rm P}} = 0$). Equations 16–18 also follow simply. For an XO F1 hybrid, all of the heterozygosity is found on the autosomes, such that $p_{12} = 1 - x$ while the hybrid index is $h = (1 \pm x)/2$, depending on which parental line contributed the X. It follows, therefore, that $2h(1-h) = (1-x^2)/2$, and $4h(1-h) - p_{12} = x(1-x)$.

2.2 Darwin's corollary (eq. 20)

For the asymmetry of log fitness between the cross directions, we will begin by assuming that the parental phenotypes – and therefore the midparental phenotype – are optimal. This means that the reciprocal XO F1, carrying different X chromosomes, will deviate in equal and opposite extents from the optimum. We will denote these deviations as $\sqrt{dn}\Upsilon$, reflecting their growth with the number of traits, and with divergence (e.g. eq. 53). The dominance deviations, which come from their shared autosomes, will be in consistent directions. As such, we can orientate the trait axes so that these deviations fall on trait 1. This means that the difference in their log fitnesses can be written as:

$$\ln w_{\mathrm{F1},♀♂} - \ln w_{\mathrm{F1},♂♀} = \left(\Delta_1 + \sqrt{dn}\Upsilon\right)^2 - \left(\Delta_1 - \sqrt{dn}\Upsilon\right)^2, \qquad \ln w_{\mathrm{MP}} = 0$$

$$= 4\sqrt{dn}\Upsilon\Delta_1 \tag{54}$$

while the mean across the cross directions yields:

$$\frac{1}{2}\left(\ln w_{\mathrm{F1},♀♂} + \ln w_{\mathrm{F1},♂♀}\right) = \frac{1}{2}\left[\left(\Delta_1 + \sqrt{dn}\Upsilon\right)^2 + \left(\Delta_1 - \sqrt{dn}\Upsilon\right)^2 + 2\sum_{i=2}^{n}\Delta_i^2\right], \qquad \ln w_{\mathrm{MP}} = 0$$

$$= dn\Upsilon^2 + \sum_{i=1}^{n}\Delta_i^2 \tag{55}$$

We now treat the dominance deviations as normal variables, such that $\Delta_i = \sqrt{d\mathrm{Var}\left(a_i\delta_i\right)}\zeta_i$, where ζ_i is a standard normal random variable, with vanishing mean and unit variance. The measure of asymmetry is therefore

$$A_{\ln w} = \frac{|\ln w_{\mathrm{F1},♀♂} - \ln w_{\mathrm{F1},♂♀}|}{\frac{1}{2}|\ln w_{\mathrm{F1},♀♂} + \ln w_{\mathrm{F1},♂♀}|} = \frac{4\sqrt{n\mathrm{Var}\left(a_1\delta_1\right)}|\zeta_1|}{\sum_{i=1}^{n} \mathrm{Var}\left(a_i\delta_i\right)\zeta_i^2 + n\Upsilon^2}$$

$$= 4\sqrt{n\Upsilon^2/V_{a\delta}}\frac{|\zeta_1|}{\sum_{i=1}^{n} \zeta_i^2 + n\Upsilon^2/V_{a\delta}}, \qquad \mathrm{Var}\left(a_i\delta_i\right) = V_{a\delta} \tag{56}$$

So this quantity is not expected to change in any consistent way with the genetic divergence, d. We have not been able to evaluate this expression analytically, but numerical integration shown in Figure S10, shows that, to a rough approximation

$$E\left(A|\Upsilon^2\right) \approx \sqrt{\frac{2}{n}}, \qquad \Upsilon^2/V_{a\delta} \sim 1 \tag{57}$$

which agrees with the simulation results reported in the main text (Figure 6c-d) and in Figure S5e-f. We can also anticipate the typical value of $\Upsilon^2/V_{a\delta}$, using eq. 16. This shows that

$$E\left(\Upsilon^2/V_{a\delta}\right) = \frac{x(1-x)}{V_\delta}, \qquad \ln w_{\mathrm{MP}} = 0 \tag{58}$$

suggesting that $\Upsilon^2/V_{a\delta} \sim 1$ will often hold for plausible parameter values.

Appendix 2: Simulation procedure

To complement our analytical predictions, we used individual-based simulations of evolutionary divergence under Fisher's geometric model. The simulation procedures were closely based on those reported in Schneemann et al. (2020), and for compatibility with earlier work, we included a constant into the log fitness function, such that:

$$\ln w = -\alpha \left\|\mathbf{z} - \mathbf{o}\right\|^2 \tag{59}$$

All simulations used $\alpha = 1/2$. In all cases, we simulated pairs of allopatric diploid populations, which were initially identical with no segregating variation. The ancestral population was phenotypically optimal for all simulations except for those presented in Figures 3e–f (modelling directional selection) and 4d (modelling locally adapted parents), where the ancestor was placed at unit distance from the optimum, such that the ancestral population had a relative fitness of $e^{-\alpha} \approx 61\%$, and the simulations began with a bout of directional selection towards the optimum. In all cases, the diverging populations were of constant size N individuals ($2N$ chromosomes), and contained simultaneous hermaphrodites with discrete non-overlapping generations.

To produce the next generation, $2N$ prospective parents were sampled with replacement, with probabilities proportional to their fitness. Gametes were produced with free recombination among all loci, and assuming infinite sites, and subject to mutation. The total number of mutations was drawn from a Poisson distribution with mean $2NU$ and distributed randomly across the $2N$ gametes. There was no back mutation. The mutation rate U was chosen such that multiple mutations generally segregated together ($NU \geq 1$). The additive effects of each mutation on each each trait, were drawn independently from a normal distribution, with vanishing means and variance denoted $V_{a_{\text{mut}}}$. We set this mutational variance at:

$$V_{a_{\text{mut}}} = -\frac{\bar{s}\Gamma\left(\frac{n}{2}\right)}{8\alpha\Gamma\left(\frac{n+2}{2}\right)} \tag{60}$$

Because the homozygous effect of a mutation was twice its additive effect, eq. 60 ensures

that the mean selection coefficient acting on a homozygous mutation occurring in an optimally fit individual was approximately $-\bar{s}$, irrespective of the number of traits under selection, n.

The heterozygous effect of a mutation was equal to its additive effect, a_{mut}, multiplied by $(1+\delta_{\text{mut}})$, where δ_{mut} is its dominance coefficient. For simulations under phenotypic additivity, we simply set $\delta_{\text{mut}} = 0$ for all mutations. With variable phenotypic dominance, the δ_{mut} were drawn from shifted beta distributions, which we bounded at -1 (for a wholly recessive mutation) and 1 (for a wholly dominant mutation).

For the simulations reported in Figures 3, 4d, 6, and S3, we used the same shifted beta distribution for all mutations, regardless of their additive effects. To do this, we set set $E(\delta_{\text{mut}}) = 0$ and $\text{Var}(\delta_{\text{mut}}) = V_{\delta_{\text{mut}}}$. These distributions are shown in Figure 3a. Generating dominance coefficients in this way allowed us to adjust the amount of variance in dominance coefficients of new mutations $V_{\delta_{\text{mut}}}$, which was reflected in V_δ.

For the simulations reported in Figures 2c, 5b and S5, we generated the δ_{mut} values to reflect some of the known properties of large and small effect mutations (Billiard et al., 2021). Specifically, small effect mutations were close to additive with little variance, whereas large effect mutations were predominantly recessive but with a higher variance, such that some would be dominant (Orr, 1991; Manna et al., 2011). This was achieved by sampling the dominance multipliers from a beta distribution such that

$$\text{E}(\delta_{\text{mut}}) \equiv \mu = 1 - \frac{2}{1 + \exp\left(-2\frac{|a_{\text{mut}}|}{\sqrt{V_{a_{\text{mut}}}}}\right)} \tag{61}$$

and

$$\text{Var}(\delta_{\text{mut}}) = \mu(\mu^2 - 1) \tag{62}$$

The distributions generated by eqs. 61–62 are illustrated in Figure S1.

For each set of parameters, we simulated 200 independent populations that each evolved until they fixed 1000 substitutions. We then randomly paired populations to obtain 100 pairs for the for-

mation of F1 hybrids. We considered the substitutions that had fixed in the number of generations it took for the first population of the pair to reach 1000 substitutions. To produce the F1, for standard biparental inheritance (and also for homogametic hybrids), we took the heterozygous effect of all substitutions. For our heterogametic hybrids, which were our example case of uniparental inheritance, we assigned a random proportion x of substitutions to be on the X chromosome, and considered their homozygous rather than their heterozygous effects. This is equivalent to a form of dosage compensation, involving up-regulation on the X (see main text). Note that these X-linked substitutions were chosen after simulating the divergence. This meant that all sites were biparentally inherited and expressed during the divergence process. This guaranteed that, for our simulations, the distribution of dominance coefficients did not differ between the X and autosomes, and both matched the mutational input, such that ($V_\delta \approx V_{\delta_{\text{mut}}}$). While this simulation procedure does not incorporate the expectation that X-linked loci will have more extreme dominance effects (Charlesworth et al., 1987), it allowed us to illustrate the conditions for Haldane's Rule, eq. 18 (see Figure 6). Furthermore, the bound on the size of dominance effects that follows from eq. 18 applies to X-linked loci, so if V_δ is smaller for autosomal loci, the bound still applies to V_δ across all sites.
